# Artificial and natural non-nutritive sweeteners drive divergent gut and genetic responses across generations

**DOI:** 10.3389/fnut.2026.1694149

**Published:** 2026-04-10

**Authors:** Francisca Concha Celume, Francisco Pérez-Bravo, Fabien Magne, Ricardo Olivares, Martin Gotteland

**Affiliations:** 1 Department of Nutrition, Faculty of Medicine, University of Chile, Santiago, Chile; 2 Department of Anthropology, Faculty of Social Sciences, University of Chile, Santiago, Chile; 3 Institute of Nutrition and Food Technology (INTA), University of Chile, Santiago, Chile; 4 Microbiology and Mycology Program, ICBM, Faculty of Medicine, University of Chile, Santiago, Chile; 5 Department of Animal Pathology, Faculty of Veterinary and Animal Sciences, University of Chile, Santiago, Chile

**Keywords:** gut microbiota, intergenerational inheritance, non-nutritive sweeteners, short chain fatty acids, *Srebp1*, stevia, sucralose, *Tlr4*

## Abstract

**Background:**

The role of non-nutritive sweeteners (NNS) in the development of metabolic alterations and chronic non-communicable diseases is controversial. It is also unclear whether these alterations are transmitted to offspring or whether the gut microbiota is involved in these processes. This study aimed to compare, in mice, the effect of parental sucralose or stevia consumption on fecal microbiota diversity/composition and short-chain fatty acid (SCFA) concentrations in mice, as well as on the expression of *Tlr4, Tnf, Tjp1* and *Srebp1* in the liver and intestines. The study also aimed to determine whether these changes are transmitted to the F1 and F2 generations.

**Methods:**

Forty-seven male and female mice were divided into three groups to receive water alone or water supplemented with sucralose or stevia (0.1 mg/ml) for 16 weeks (F0 generation). The F0 mice were then bred to produce the F1 generation, and the F1 mice were bred to produce the F2 generation. The F1 and F2 animals did not receive NNS.

**Results:**

No changes in the glucose oral tolerance test were observed between in the F0 generation, while the glycemic response was mildly altered in the F1 and F2 male mice in the Sucralose group. Compositional changes in the fecal microbiota were greater in the F0 and F1 generations, particularly the Sucralose group. Animals from the F0 Sucralose and Stevia groups had lower SCFA concentrations, and this trait was passed on to next generations. In terms of gene expression, *Tlr4* and *Tnf* were overexpressed in the intestine of the F0/F1 Sucralose group, while Srebp1 expression was lower in the liver of the F0 Sucralose group, a change that persisted in the F1 and F2 generations. *Tlr4* and *Tnf* expression was higher in the F1 Stevia group and normalized in the F2.

**Conclusion:**

Sucralose consumption affects glucose tolerance, the expression of liver *Srebp1* and intestinal *Tnf* and *Tlr4*, fecal microbiota composition and SCFA concentrations, and these changes are transmitted across generations. The effects of stevia are mainly observed in the F1 generation.

## Introduction

High-sugar diets have been shown to promote hyperinsulinemia, impaired glucose tolerance, and adiposity, and to be associated with an increased risk of chronic non-communicable diseases (NCDs) in both adults and children (1–4). Non-nutritive sweeteners (NNS), including acesulfame K, aspartame, cyclamate, saccharin, sucralose, and steviol glycosides (stevia), are food additives that are widely used as sugar substitutes to provide a sweet taste without calories. Originally developed to mitigate the metabolic consequences of excessive sugar intake and to facilitate weight management (5), the consumption of NNS has increased worldwide across all age groups, including among women of childbearing age (6). For example, a recent national consumer survey in the United States reported that over 140 million Americans used NNS in 2020 (7). In Chile, one of the countries with the highest obesity rates, we recently observed that a higher proportion of foods and beverages containing NNS were available on the local market, compared to other countries, making it challenging for consumers to find NNS-free products in some food categories (8). In this context, it is noteworthy that the World Health Organization (WHO) recently published a guideline questioning the long-term benefits of NNS and suggesting that they do not aid in weight control and may be associated with potential adverse long-term effects, including an increased risk of type 2 diabetes (T2D) and cardiovascular disease (9, 10).

The mechanisms underlying these adverse metabolic effects involve different pathways that have been extensively investigated. Animal studies suggest that, by stimulating oral sweet taste receptors (T1R2/T1R3) without bringing calories, NNSs could interfere with learned responses that contribute to glycemic control and energy homeostasis, affecting incretin and neuromediator release, which can lead to compensatory increases in appetite and energy intake (11–14). In the intestine, NNSs stimulate sweet taste receptors on enterocytes and enteroendocrine cells, promoting postprandial glucose uptake by active (SGLT1) and/or facilitated (GLUT2) transporters (15, 16). Such impairment in glycemic control would be amplified by the deleterious effects of NNS on the composition, diversity, and function of the gut microbiota, which mainly results in lower production of short chain fatty acids (SCFAs) and increased microbiota virulence potential (17–19). Indeed, decreased SCFA levels would promote glucose intolerance by impairing insulin sensitivity, reducing incretin secretion, and increasing inflammation and oxidative stress (20, 21), therefore amplifying the direct effect of NNS on these outcomes (22, 23). To summarize, NNSs such as sucralose can disturb glucose and energetic metabolism directly through their pro-oxidant and pro-inflammatory effects or indirectly through the stimulation of sweet taste receptors in different tissues, and the alterations of gut microbiota composition and SCFA production. The impact of NNS on gut microbiota is, however, controversial, with some studies reporting negative alterations, while others suggest neutral or even beneficial effects, underscoring the complexity of these interactions (24).

Despite their widespread use by women of childbearing age, the effects of parental consumption on offspring remain understudied. Maternal consumption of NNS during pregnancy and lactation has been associated with increased risk of preterm birth and metabolic disturbances in childhood (25). Among the changes reported in animals consuming NNS, such as increased weight gain, low-grade inflammation, decreased insulin sensitivity, and impaired functions of the gut microbiota, intestinal barrier, and liver, some can be transmitted to offspring (26–29). However, the mechanisms involved in this transmission are not well understood and may include mother-to-child transfer of altered maternal microbiota with lesser capacity of SCFA production. Beyond the negative impact described above for decreased SCFA levels, it is interesting to note that some SCFAs mainly butyrate and propionate, can inhibit histone deacetylase (HDAC) expression and/or activity, thereby increasing histone acetylation and gene expression (30). Therefore, maternal intake of NNS could indirectly affect offspring health through the transmission of dysbiotic microbiota associated with epigenetic mechanisms.

The current work is part of a larger study seeking to evaluate the intergenerational effect of parental (F0) consumption of sucralose or stevia in mice on various factors including (1) anthropometric and metabolic parameters, (2) expression of biomarkers of inflammation, metabolism and barrier function in the intestine, liver, adipose tissue and muscle, and (3) fecal microbiota diversity/composition and SCFA concentrations, in the first (F1) and second (F2) generations of animals not directly exposed to these NNS. Part of the results has been recently published (31), indicating that sucralose or stevia does not affect body weight gain in the F0 generation but increases it in the F1 generation. In addition, they also show that NNS consumption differentially affects *Hdac3* expression at the hepatic and intestinal levels, with a decreased expression in the liver and an increased expression in the intestine of F0 mice. Although the hepatic expression of *Hdac3* was normalized to the control values in the F1 and F2 animals of both NNS groups, its intestinal expression remained higher in the F1 generations of both the sucralose and stevia groups and was subsequently normalized in the F2 generation. Therefore, the changes in hepatic expression of *Hdac3* induced by parental consumption of NNS were not transmitted to the F1 and F2 generations, whereas those in intestinal expression were enhanced in the F1 and attenuated in the F2 generation (31).

In the current manuscript, we describe how parental (F0) sucralose or stevia consumption influences glucose tolerance, the liver or intestinal expression of genes involved in inflammation (*Tlr4* and *Tnf* ), gut barrier function (*Tjp1*) and metabolism (*Srebp1*), as well as fecal microbiota (FM) diversity/composition, fecal concentrations of SCFA, and the transmission of these changes to the F1 and F2 generations.

## Material and methods

### Animals and diet

Forty-seven male and female C57BL/6J mice were bred in the animal facilities of the Institute of Nutrition and Food Technology (INTA), University of Chile, according to the animal care and handling protocol approved by the Institutional Animal Care and Use Committee (PT2021-01-MG-FC). The animals were maintained at 20–25 °C with a 12-h light/dark cycle. At 4 weeks of age, unrelated, primiparous female and male mice (F0, parental generation) were randomized into 3 groups to receive water alone (Control) or water supplemented with sucralose (Sucralose group) or stevia (Stevia group) for 16 weeks. The concentration of sucralose and stevia was 0.1 mg/ml, approximately 5–15 mg/kg body weight/day, which is equivalent to the FDA-approved acceptable daily intake (ADI) for humans (15 and 4 mg/kg/day, respectively) and previously used in other studies (19). The animals had *ad libitum* access to water and a standard chow diet (Prolab RMH 3000 5P00, USA) providing approximately 22% protein, 5% fat, and 15.4% neutral detergent fiber, with ~60% of total calories derived from carbohydrates (4.17 kcal/g). All animals across F0, F1, and F2 generations received the same diet throughout the study.

**Figure 1:**
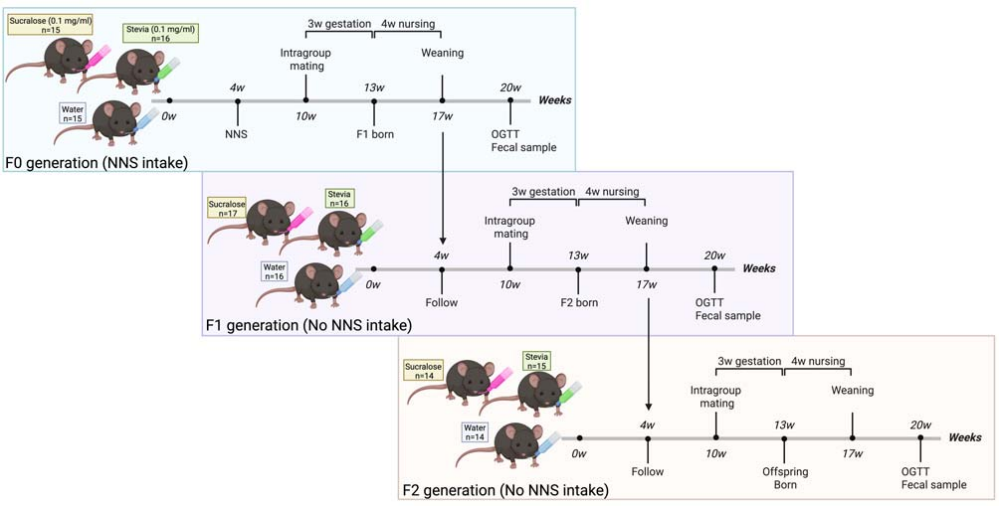
Experimental design for intergenerational exposure to sucralose and stevia. At 4 weeks of age, non-consanguineous, primiparous female and male C57BL/6J mice [parental (F0) generation] were randomly distributed into three groups to receive water alone (control) or water supplemented with sucralose (0.1 mg/ml) or stevia (0.1 mg/ml) for 16 weeks. To assess the intergenerational effect of non-nutritive sweeteners (NNSs), F0 mice belonging to the same treatment group were mated at 10 weeks of age. After 4 weeks of lactation, the F1 offspring were weaned and followed until 20 weeks of age. F1 and F2 generations did not receive direct NNS exposure. All mice were maintained under identical housing and husbandry conditions, including the same animal room, cage type and bedding, chow batch, water source and bottle cleaning protocol, and standardized cage cleaning schedule.

The experimental design of the study is described in Figure 1. To assess the intergenerational effect of NNS, mice from the same group were crossed at week 6 of treatment. After 4 weeks of lactation, the first generation (F1) was weaned and followed the same protocol as F0. Unrelated F1 animals from the same group were crossed to generate the second generation (F2). F1 and F2 mice received only pure water (without NNS) and standard chow.

Changes in body weight and food and water consumption were measured weekly, the corresponding results have been previously reported (31). At week 20, the F0, F1, and F2 mice were euthanized using deep anesthesia induced by the inhalation of 5% isoflurane (Zoetis, Parsippany, NJ, USA) in a one-liter glass tank, as described by Schmid et al. (32). Ileal and hepatic tissue samples were collected, snap-frozen in liquid nitrogen, and stored at −80 °C.

### Oral glucose tolerance test (OGTT)

Prior to euthanasia, mice were fasted for 6 h and subjected to an OGTT using 1.5 g of glucose per kg of body weight. Blood samples were collected at 0, 15, 30, 60, and 120 min, and blood glucose levels were measured using an Accu-Chek Performa glucometer (Roche Diagnostics, Mannheim, Germany).

### Fecal microbiota analysis

Fecal samples were collected from a subgroup of mice (six animals per group and generation) before euthanasia and stored at −80 °C until processing. Bacterial DNA was extracted using the PowerSoil DNA Isolation Kit (MOBIO laboratories, Qiagen, Germany) according to the manufacturer's instructions, including a bead-beating step to ensure efficient lysis of Gram-positive bacteria. DNA was quantified using a Tecan Infinite M200 Pro (Tecan, Austria) and DNA integrity was assessed by 1% agarose gel electrophoresis. PCR amplification of the “v3-v4” hypervariable region of the bacterial 16S rRNA gene (33) (Supplementary Table S1) was performed using barcoded primers, followed by library preparation, and paired-end amplicon sequencing on the Illumina MiSeq platform (Illumina Inc, San Diego, CA, USA). Sequencing services were provided by Omega Bioservices (Norcross, USA). Negative extraction controls and PCR no-template controls were included throughout DNA extraction, amplification, and library preparation steps to monitor potential reagent and laboratory contamination.

Demultiplexed FASTQ files were processed using the DADA2 pipeline (v1.x) to perform denoising, paired-end read merging, and removal of chimeric sequences, resulting in high-resolution amplicon sequence variants (ASVs). More specifically, quality-filtering was performed using the *filterAndTrim* function, with reads truncated at 280 bp (forward) and 250 bp (reverse), primer sequences (20 bp from each end) removed, a maximum expected error threshold of two for forward reads and five for reverse reads, and truncation at a quality score of two. Reads containing ambiguous bases were discarded, and PhiX contaminant sequences were removed. Error rates were learned separately for forward and reverse reads; dereplicated reads were denoised using the DADA algorithm, and paired reads were merged requiring a minimum overlap of 15 bp and allowing a maximum of one mismatch.

The full ASV table generated from the study was imported into the phyloseq R Package (v.1.42.0) for downstream analysis. To reduce spurious and low-abundance features, taxa were first filtered by prevalence, retaining only those with a total abundance greater than 100 reads across all samples. Non-bacterial and host-associated sequences were removed by excluding taxa annotated as Eukaryota, Chloroplast, or Mitochondria at any taxonomic rank (Kingdom to Genus). Taxa lacking phylum-level annotations were also excluded. DADA2-processed data were not rarefied prior to analysis. Because denoising pipelines remove singletons and explicitly model sequencing errors, richness estimates and rarefaction curves are highly sensitive to bioinformatic parameters rather than biological variations. In this context, rarefaction does not adequately correct for sequencing depth biases and may produce misleading diversity estimates. Moreover, rarefaction discards valid reads, increasing measurement error and reducing statistical power. Given that ASV tables are inherently compositional, DADA2 output is more appropriately analyzed using depth-aware or log-ratio–based methods that retain all reads, rather than equalizing library sizes through rarefaction (34).

Taxonomy assignment was performed using the SILVA reference database (version 138) with a 99% similarity threshold. All abundance, diversity, and ordination analyses were conducted using the Phyloseq package (35). Alpha-diversity was assessed using the Shannon index and Observed species richness calculated from non-rarefied ASV tables Beta diversity was calculated using Bray–Curtis dissimilarity matrices, and intergroup and intergenerational comparisons were visualized using Canonical Correspondence Analysis (CCA) with 95% confidence ellipses. Statistical differences in community composition were evaluated using permutational multivariate analysis of variance (ADONIS). Differentially abundant taxa within each generation and treatment group were identified using Linear Discriminant Analysis Effect Size (LEfSe) (36). A Kruskal-Wallis test with Bonferroni correction was applied, using a significance threshold of *p* < 0.05, and an effect size threshold of 2.0 on the logarithmic LDA score to discriminate biologically relevant taxa.

### Determination of fecal short-chain fatty acids

Fecal SCFAs were determined as previously described by Cires et al. (37). Frozen fecal samples (40–100 mg) were thawed and homogenized with 500 μl of distilled water, pH adjusted to 2–3 with 0.68 M HCl and incubated for 10 min at room temperature with occasional agitation. After centrifugation (10 min, 14.000 rpm, room temperature), 2-ethylbutyric acid was added to the supernatant as an internal standard at a final concentration of 1 mM. Detection and quantification of SCFAs were performed by gas chromatography on an Agilent 7890A instrument (Agilent, USA), equipped with a flame ionization detector (FID) and a capillary column (Resteck Stabilwax-DA, Resteck, USA) of 30 m length, 320 μm inner diameter and coated with a 1 μm film. Separation of SCFAs was performed over a temperature range of 120–240 °C using hydrogen as a carrier gas (6 ml/min). Injector and detector temperatures were set at 240 and 265 °C, respectively. A SCFA standard (Restek, USA) containing acetic, propionic, butyric, valeric, isobutyric, and isovaleric acids at the same concentration (1 μg/ml), was processed in the same conditions and used for calibration. Results were expressed as mmol/g of stool.

### RNA extraction, quantitative PCR, and gene expression

Total RNA was extracted from liver and intestinal tissues using the Total RNA kit I (Omega Bio-Tek, Norcross, GA, USA) and converted to cDNA using the High-Capacity cDNA Reverse Transcription Kit (Applied Biosystems, Waltham, MA, USA) according to the manufacturer's instructions. The quality of the cDNA was evaluated based on the 260/280 absorbance ratio on the Tecan Infinite M200 Pro instrument (Tecan, Austria). The cDNA samples were stored at −20 °C until use. Real-time PCR was performed with Brilliant II SYBR Green QPCR Master Mix (Applied Biosystems, Waltham, Massachusetts, USA) according to the manufacturer's instructions. Gene expression of sterol regulatory element-binding protein 1 (*Srebp1*), toll like receptor-4 (*Tlr4*), tumor necrosis Factor (*Tnf* ), and *zonula occludens* 1 (*Tjp1*) was determined by RT-PCR, using β-actin as housekeeping gene (38–42) (Supplementary Table S1) because its expression was shown to be stable across all the experimental groups, generations, and tissues analyzed, with minimal variability between samples. Amplification was performed on the AriaMx real-time PCR system (Agilent Technologies). Changes in gene expression were calculated using the primer-efficiency-corrected method (43).

### Statistical analysis

Statistical analyses were performed using SPSS version 27.0 (IBM SPSS, Chicago, IL, USA), GraphPad Prism 9.5.1 (GraphPad Software, LLC, Boston, USA) for data visualization, and the R software package (version 4.1.0) for microbiota. Given the nonparametric distribution of the data (assessed by Shapiro-Wilk test), results were expressed as median ± interquartile range. Intergenerational and intergroup differences were compared using the nonparametric Kruskal-Wallis test with *post-hoc* Dunn test with Bonferroni corrections for multiple comparisons. For the analysis of gene expression, fecal microbiota diversity and composition, and fecal SCFAs, no significant differences were observed between male and female mice, and data from both sexes were therefore pooled for statistical analysis. Significance was determined at *p* < 0.05.

## Results

### Parental sucralose consumption is associated with mild blood glucose changes in offspring, primarily in males

The effects of sucralose and stevia consumption on glucose metabolism were assessed using an oral glucose tolerance test (OGTT). As shown in Supplementary Figures S1A, B, G, H, OGTT glucose levels and AUC_Glc_ in the F0 generation did not differ between the Sucralose and Control groups in both males and females. However, males in the Stevia group had lower glucose at 120 min compared to Control and Sucralose groups (*p* = 0.004 and *p* = 0.010, respectively), without differences in AUC_Glc_ (Supplementary Figures S1G, H). F1 males in the Sucralose group had lower glucose at 120 min than the Control and Stevia groups (*p* = 0.021 and *p* = 0.003, respectively; Supplementary Figures S1C, D), but had higher AUC_Glc_ than the Control group (*p* = 0.005), while no changes were observed in females (Supplementary Figures S1G, H). In the F2 generation (Supplementary Figures S1E–H), fasting glycemia was higher in females from the Stevia group (*p* = 0.035). Males from the Sucralose group males showed higher fasting glucose than Control (*p* = 0.026) and lower glycaemia at 120 min than the Stevia group (*p* = 0.023), with no changes in the other variables.

No intergenerational changes in glycemia were observed between the F0, F1, and F2 generations of the control animals, both in males and females (Supplementary Figures S2A, B, G, H). In the Sucralose group, F2 males had higher basal glycemia than F1 (*p* = 0.022; Supplementary Figures S2C, D, G, H). In the Stevia group, basal glycemia in F2 females was higher than F1 (*p* = 0.017), and F1 and F2 males showed higher glucose at 120 min than F0 (*p* = 0.001 and *p* = 0.004, respectively; Supplementary Figures S2E–H).

### Parental consumption of sucralose or stevia is associated with alterations in *Tnf, Tlr4*, and *Srebp1* expression across generations

We determined the effect of the parental consumption of NNS on the mRNA expression of *Tjp1, Tlr4, Tnf* and *Srebp1* in liver and/or intestinal tissues by RT-PCR in the 3 generations. These biomarkers were selected as they capture the key mechanistic links between gut-derived inflammation, intestinal barrier integrity, innate immune activation, and metabolic dysregulation, all pathways repeatedly implicated in NNS-associated metabolic effects. The *Tjp1* gene encodes the protein *zonula occludens*-1 (ZO-1), which is involved in the formation of tight junctions and the regulation of the barrier function in the intestinal epithelium. Impaired gut barrier facilitates LPS translocation to plasma and the subsequent activation of toll-like receptor-4 (TLR-4), a pattern recognition receptor, and the production of pro-inflammatory TNF. As a consequence, chronic inflammation develops accompanied by metabolic dysfunction, reflected by changes in sterol regulatory element-binding protein-1 (SREBP-1) that plays a key role in the induction of lipogenesis in the liver and the regulation of glucose metabolism.

**Figure 2:**
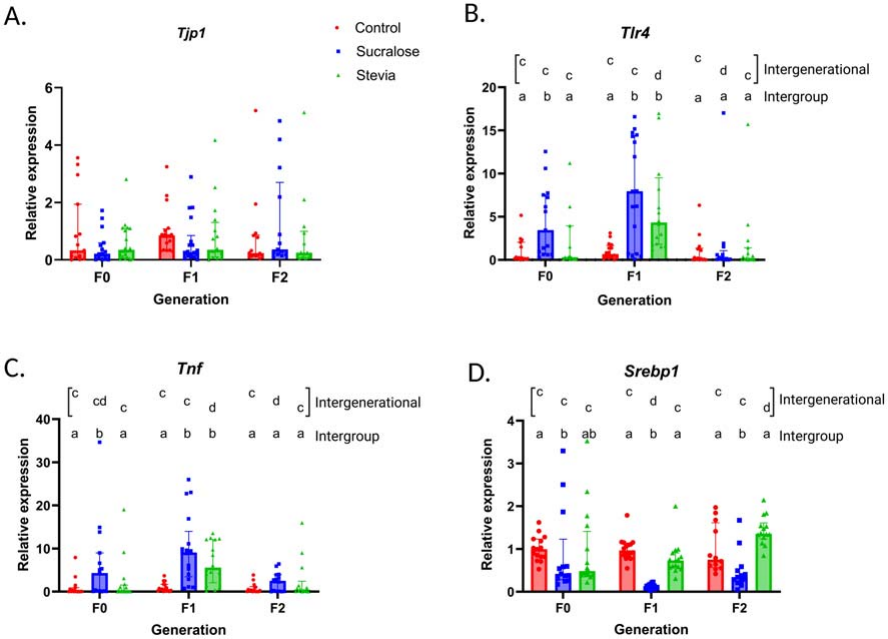
Expression of intestinal *Tjp1, Tnf* , and *Tlr4*, and liver *Srebp1* in the generations F0, F1, and F2 of mice from the Control, Sucralose and Stevia groups. Intergroup and intergenerational intestinal expression of *Tjp1* gene mRNA **(A)**, *Tlr4* gene mRNA **(B)**, and *Tnf* gene mRNA **(C)**, and liver expression of *Srebp1* gene mRNA **(D)**. (*n* = 12–17 animals/group). Data were analyzed using the Kruskal-Wallis test, with multiple comparisons carried out using Dunn's test adjusted with Bonferroni correction. Results are expressed as median ± interquartile range. Different letters indicate statistical significance, *p* < 0.05. The first line with letters a and b indicates statistical significances when comparing different treatment groups by generation. The second line with letters c and d indicates statistical significances when comparing different generation by treatment group.

Results from Figure 2A indicate that intestinal *Tjp1* expression was not affected by NNS consumption in the F0 generation, nor in the offspring. Sucralose intake was associated with higher intestinal *Tlr4* and *Tnf* expression (*p* = 0.006 and *p* = 0.02, respectively; Figures 2B, C) and reduced liver expression of *Srebp1* (*p* = 0.03; Figure 2D) in the F0 animals, compared to controls. No changes were observed in the Stevia group, compared to the Control group, and *Tlr4* and *Tnf* expression were lower than in the Sucralose group (Figures 2B, D). In the F1 generation, intestinal *Tlr4* and *Tnf* were overexpressed in both Sucralose and Stevia groups, compared to controls (*p* = 0.005 and *p* = 0.001 for both groups, respectively; Figures 2B, C), and these changes normalized in F2. Liver *Srebp1* expression remained lower in the Sucralose in the F1 and F2 generations, compared to the Control (*p* = 0.001 in F1 and *p* = 0.02 in F2) and Stevia groups (*p* = 0.001 in F1 and *p* = 0.001 in F2; Figure 2D).

When comparing each treatment between generations, sucralose-associated *Tlr4* and *Tnf* overexpression persisted in the F1 (*p* = 0.03 and *p* = 0.001, respectively, Figures 2B, C). In the F2 generation, *Tlr4* expression was attenuated (*p* = 0.03 Figure 2B) while that of *Tnf* was similar to F0 and lower than F1 (*p* = 0.03 Figure 2C). In the stevia group, the expression of these genes increased in F1 (*p* < 0.001 for *Tlr4* and *p* = 0.003 for *Tnf* Figures 2B, C), and was normalized in F2, with respect to F0, being significantly lower than in F1 (*p* = 0.001 and *p* = 0.02, respectively; Figures 2B, C). The liver expression of *Srebp1* in the Sucralose group decreased in the F1 generation, compared to the F0 (*p* = 0.001 Figure 2D), while in the Stevia group, it was significantly higher in the F2 generation than in the F0 and F1 generations (*p* = 0.002 and *p* = 0.008, respectively; Figure 2D).

Taken together, these results suggest that NNS consumption, particularly sucralose, is associated with changes in gene expression in the intestine and liver, with these effects persisting in the F1 generation and being attenuated in the F2 generation.

### Microbiota analysis

Across fecal samples, sequencing generated an average of 119,610 ± 68,478 raw paired-end reads per sample. Following quality filtering and primer trimming, 70,251 ± 37,082 reads were retained. Paired-end merging yielded 62,276 ± 36,382 reads, and subsequent chimera removal resulted in 58,940 ± 34,297 high-quality non-chimeric reads per sample, corresponding to an overall retention of approximately 49% of the initial reads. Negative control samples yielded substantially fewer reads at all stages of the DADA2 pipeline. Controls contained 1,497 ± 1,786 raw reads, of which 94 ± 83 reads remained after quality filtering. All merged control reads passed chimera removal, resulting in 15 ± 21 final non-chimeric reads, indicating minimal background amplification and low levels of contamination. Given the low read counts observed in negative controls and the robust sequencing depth across fecal samples, no additional filtering or exclusion of fecal samples was required, and all samples were retained for downstream analyses.

### Parental consumption of NNS is associated with changes in microbiota diversity, which are transmitted to the F1 and F2 generations

**Figure 3:**
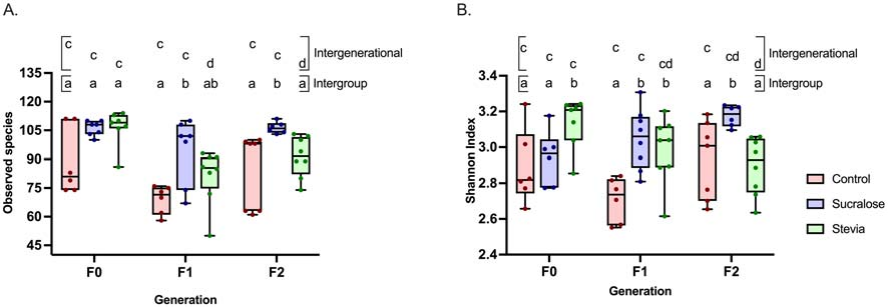
Intraindividual diversity of the fecal microbial of mice from the Control, Sucralose and Stevia groups in the generations F0, F1, and F2. Intraindividual diversity was determined through the number of observed species **(A)** and the Shannon index **(B)**. Data were analyzed using the Kruskal-Wallis test, with multiple comparisons carried out using Dunn's test adjusted with Bonferroni correction. Results are expressed as median, interquartile range, and minimum and maximum (*n* = 6 samples/group). Different letters indicate statistical significance, *p* < 0,05. The first line with letters a and b indicates statistical significances when comparing different treatment groups by generation. The second line with letters c and d indicates statistical significances when comparing different generations by treatment group.

**Figure 4:**
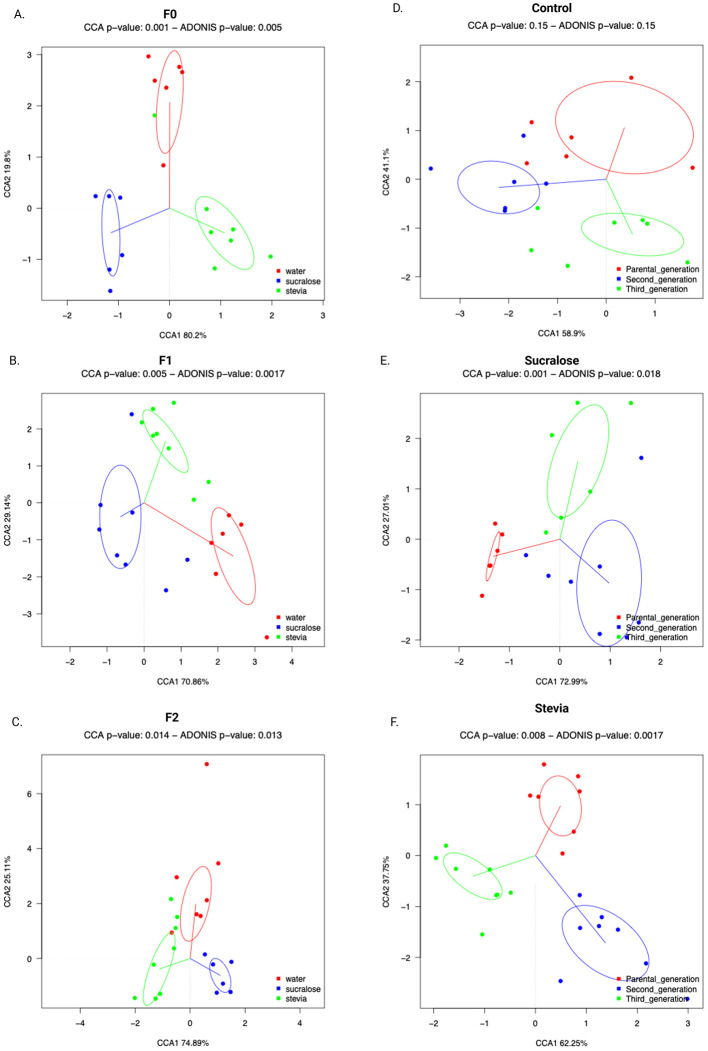
Interindividual microbial diversity of the fecal microbial of mice from the Control, Sucralose and Stevia groups in the generations F0, F1, and F2. Beta diversity was based on weighted Unifrac distances, with ellipses representing the 95% confidence interval for each group (*n* = 6 samples/group). The beta diversity corresponding to the generations F0, F1, and F2 is shown in **(A–C)**, respectively. The beta diversity corresponding to the groups Control, Sucralose and Stevia is shown in **(D–F)**, respectively. Data were analyzed using the ADONIS test (*p* < 0.05).

Fecal microbiota (FM) composition was assessed by sequencing the V3-V4 region of the 16S rRNA gene of fecal bacterial DNA. In the F0 generation, α-diversity, as measured by the number of observed species, did not differ between groups (Figure 3A), while the Shannon index was higher in the Stevia group than in the Control and Sucralose groups (*p* = 0.038 and *p* = 0.042, respectively; Figure 3B). β-Diversity, based on Bray-Curtis distances, showed significant differences in FM composition among all groups (*p* = 0.006; Figure 4A).

In the F1 generation, the Sucralose group exhibited more observed species than the Control group (*p* = 0.0018; Figure 3A), and the Shannon index was higher in both the Sucralose and Stevia groups compared to the Control group (*p* = 0.001 and *p* = 0.005, respectively; Figure 3B). The β-diversity also differed between groups in this generation (*p* = 0.002; Figure 4B). In the F2 generation, the Sucralose group showed higher observed species and Shannon index compared to Control (*p* = 0.02 for both) and Stevia groups (*p* = 0.004 and *p* = 0.001, respectively; Figures 3A, B), and the differences in β-diversity persisted in this generation (*p* = 0.013; Figure 4C).

When comparing across generations, no changes in α- or β-diversity were observed in the Control group (Figures 3A, B, 4D). In the Sucralose group, the number of observed species did not change across generations, but the Shannon index was higher in F2 compared to F0 (*p* = 0.01; Figure 3B), and significant differences in β-diversity were also observed between generations (*p* = 0.018; Figure 4E). In the Stevia group, the number of observed species decreased in F1 and F2 compared to F0 (*p* = 0.001 and *p* = 0.01, respectively; Figure 3A), and the Shannon index was lower in the F2 than in the F0 (*p* = 0.009; Figure 3B). As in the Sucralose group, β-diversity differed between generations (*p* = 0.0017; Figure 4F).

### Parental consumption of NNS is associated with changes in fecal microbiota composition in the F0, F1, and F2 generations

**Figure 5:**
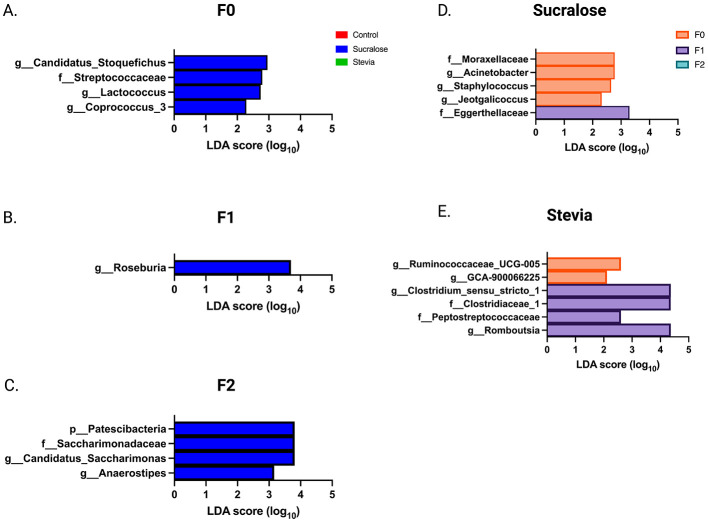
Linear discriminant analysis (LDA) determined by LEfSE for each treatment group and generation. Taxonomic groups showing LDA scores >2.0 with *p* < 0.05 at the phylum, family, and genus levels are shown. **(A–C)** show the LEfSe analysis for generations F0, F1, and F2, respectively. LEfSE at the intergenerational level for the sucralose and stevia group is shown in **(D, E)**. (*n* = 6 animals/group).

The analysis of FM in all mice identified nine phyla, 51 families, and 126 genera. The core microbiota at the genus level (i.e. the genera present in 95% of all animals) is described in Supplementary Table S2. The FM composition of the F0 generation showed significant changes in the NNS groups compared to the controls (Supplementary Table S3). In the Sucralose group, 17 genera differed from the control group, of which five belong to the core microbiota, with higher abundances of *Candidatus_Stoquefichus, Coprococcus*_3, *Jeotgalicoccus, Acinetobacter, Lactococcus, Ureaplasma, Streptococcus, Desulfovibrio, Bilophila, Staphylococcus* and *Lactobacillus*, and lower abundances of *Ruminiclostridium*_9, *Parabacteroides*, Lachnospiraceae_FCS020_group, Ruminococcaceae-_UCG−014, *Romboutsia*, and *Oscillibacter*. In contrast, the Stevia group showed changes in only four genera: *Erysipelatoclostridium, Harryflintia, Acetatifactor, and Clostridium* sensu stricto 1, none of which were from the core microbiota. In addition, the abundance of 17 taxa differed between both the sucralose and Stevia groups. LEfSe analysis revealed enrichment in Streptococcaceae and *Coprococcus*_3, *Lactococcus* and *Candidatus_Stoquefichus* genera in the Sucralose group (Figure 5A).

In F1 animals (Supplementary Table S4), the Sucralose group showed significant differences compared to the controls, with two phyla and 15 genera affected, 10 of which were part of the core microbiota. The Sucralose group exhibited higher abundances of Patescibacteria, *Lactobacillus, Alloprevotella, Streptococcus, Candidatus_ Saccharimonas, Enterorhabdus, Candidatus_Stoquefichus, Desulfovibrio, Erysipelatoclostridium, Roseburia, Ureaplasma* and *Peptococcus*, and lower abundances of Cyanobacteria, Prevotellaceae_Ga6A1_group, Prevotellaceae_UCG−001, *Oscillibacter*, and *Bifidobacterium*. The Stevia group showed changes in only nine genera, none of which belonged to the core microbiota. Specifically, there was a higher abundance of Deferribacteres, *Lactobacillus, Alloprevotella, Dubosiella, Mucispirillum, Parasutterella* and *Clostridium*_Sensu_Stricto_1, and a lower abundance of *Candidatus_Soleaferrea* and Lachnospiraceae_NK4A136_group. As in the F0 generation, *Rikenella, Candidatus_Arthromitus, Anaerotruncus, Odoribacter*, GCA−900066225, *Intestinimonas* and DNF00809 were more abundant in the Sucralose group than in the Stevia group. Consistent with the F0 generation, sucralose exposure altered the abundance of a greater number of taxa than stevia, and the LEfSe analysis showed an enrichment in the genus *Roseburia* in this group (Figure 5B).

In F2 animals (Supplementary Table S5), 13 taxa in the Sucralose group differed from the Control group, of which seven belonged to the core microbiota, with higher abundance of Patescibacteria, *Turicibacter, Butyricicoccus, Intestinimonas* GCA−900066225, *Ureaplasma, Streptococcus, Aanaerostipes*, Ca*ndidatus Saccharimonas*, and *Roseburia*, and lower abundance of Bacteroidetes, *Muribaculum*, and Lachnospiraceae_UCG−006. The Stevia group showed changes in five taxa, *Turicibacter*, which was more abundant, and Cyanobacteria, *Rikenella, Ruminococcus*_1, and *Clostridium*_Sensu_Stricto_1, which were less abundant, with none of these taxa belonging to the core microbiota. The differences between the Sucralose and Stevia groups reported in the previous generations persisted in the F2, affecting 13 taxa. LEfSE analysis showed that Sucralose continued to induce greater changes compared to stevia, with enrichment in Patescibacteria, Saccharimonadaceae, *Anaerostipes* and *Candidatus_Saccharimonas* (Figure 5C).

Taken together, these results indicate that NNS-induced changes in FM composition are greater in the F0 and F1 generations and more intense in the Sucralose group than in the Stevia group.

### NNS-associated changes in fecal microbiota composition of the F0 generation are inherited in the offspring

Intergenerational changes in FM composition were also evaluated. In the Sucralose group (Supplementary Table S6), 21 taxa differed between F0 and F1. Eight of them (38.1%) remained altered in F2 while the other 13 taxa returned to their F0 baseline value. LEfSe analysis suggests that the F1 and F2 animals were depleted in Moraxellaceae, *Jeotgalicoccus, Staphylococcus* and *Acinetobacter*, while F1 animals were enriched in Eggerthellaceae (Figure 5D). When the Sucralose groups of the three generations were combined, a positive Pearson correlation was observed between the relative abundance of Patescibacteria, Saccharimonadaceae, and *Candidatus*_*Saccharimonas* and *Tlr4* expression (*r* = 0.75, *p* = 0.00015, for each).

In the Stevia group (Supplementary Table S7), 11 taxa differed between F0 and F1. Six of them (54.5%) remained altered in F2 while the other five taxa returned to their F0 baseline value. In addition, five taxa became different from F0 only in the F2 generation. LEfSe analysis showed a depletion in Ruminococcaceae_UCG−005 and GCA−900066225 in F1 and F2 animals, while Clostridiaceae_1, Peptostreptococcaceae and the genera *Romboutsia* and *Clostridium*_Sensu_Stricto_1 were enriched in F1 (Figure 5E). The percentage of taxa that changed between F0 and F1, F1 and F2, and the taxa that returned to their baseline F0 value in the F2 did not differ between the Sucralose and Stevia groups (not significant by chi-square).

Taken together, these results show that NNS-associated changes in FM are transmitted to the first generations and partially restored in the second generation.

### Parental consumption of NNS is associated with changes in fecal SCFA concentrations in the F0, F1, and F2 generations

**Figure 6:**
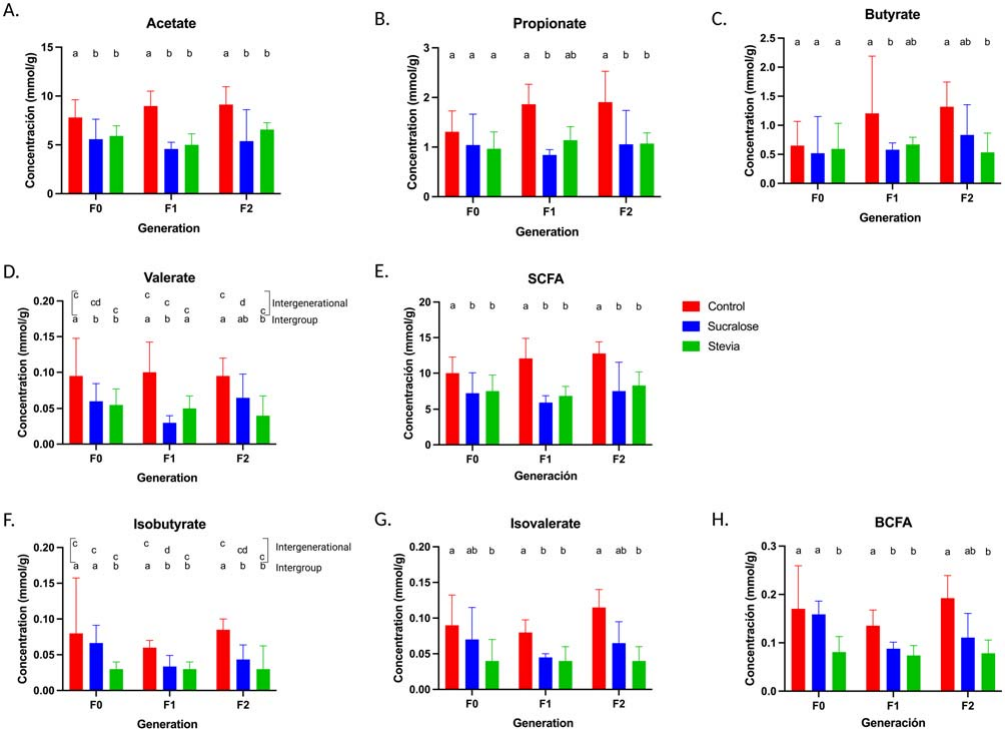
Fecal concentrations of short chain fatty acids (SCFA) and branched SCFA (BSCFA) in mice from the Control, Sucralose and Stevia groups in the generations F0, F1, and F2. Fecal concentration of acetate **(A)**, propionate **(B)**, butyrate **(C)**, valerate **(D)**, and total fecal SCFA **(E)**. Fecal concentration of isobutyrate **(F)**, isovalerate **(G)**, and total fecal BSCFA **(H)**. (*n* = 12–17 animals/group). Data were analyzed using the Kruskal-Wallis test, with multiple comparisons carried out using Dunn's test adjusted with Bonferroni correction. Results are expressed as median ± interquartile range. Different letters indicate statistical significance, *p* < 0.05. The first line with letters a and b indicates statistical significances when comparing different treatment groups by generation. The second line with letters c and d indicates statistical significances when comparing different generations by treatment group.

Quantification of fecal SCFAs and branched SCFAs (BSCFAs) was performed by gas chromatography. In F0 animals, both Sucralose and Stevia groups showed reduced acetate and valerate concentrations compared to controls (*p* = 0.05 and *p* = 0.02, respectively; Figures 6A, D), while no differences were observed in propionate and butyrate (Figures 6B, C) or in SCFA proportions (Supplementary Table S8). Total SCFA (excluding BSCFA) concentrations were lower in the Sucralose and Stevia groups than in controls (*p* = 0.05 and *p* = 0.02, respectively; Figure 6E), while the proportion of total SCFA was higher in the Stevia group than in the Control and Sucralose groups (*p* = 0.002 for both; Supplementary Table S8).

In the F1 generation, acetate, propionate, butyrate, and valerate concentrations were significantly lower in the Sucralose group, compared to controls (*p* = 0.001, *p* = 0.001, *p* = 0.009, and *p* = 0.001, respectively; Figures 6A, D), whereas only acetate was lower in the Stevia group (*p* = 0.003; Figure 6A). No changes in the proportions of these SCFAs were observed between the Sucralose and Control groups. However, the Stevia group had higher proportions of propionate, valerate, and total SCFA compared to Sucralose group (*p* = 0.009, *p* = 0.01, and *p* = 0.009, respectively; Supplementary Table S8). The concentration of total SCFAs was lower in the Sucralose and Stevia groups than in the Control group (*p* = 0.001 and *p* = 0.006, respectively Figure 6E).

In the F2 generation, the Sucralose group had lower acetate and propionate concentrations compared to controls (*p* = 0.01 and *p* = 0.006, respectively; Figures 6A, B), while acetate, propionate, butyrate, and valerate concentrations were reduced in the Stevia group (*p* = 0.02, *p* = 0.001, *p* = 0.01, and *p* = 0.004, respectively; Figures 6A–D). Total SCFA concentrations remained lower in both NNS groups compared to controls (*p* = 0.008 and *p* = 0.006, respectively; Figure 6E). However, the Stevia group had higher total SCFA proportions than the Control (*p* = 0.006; Supplementary Table S8).

Intergenerational analysis revealed no significant differences in SCFA levels in the Control or Stevia groups across generations. However, valerate concentrations were lower in F1 compared to F2 in the Sucralose group (*p* = 0.002; Figure 6D), with a reduction in valerate proportions compared to F0 and F2 (*p* = 0.02 and *p* = 0.03, respectively; Supplementary Table S9).

### NNS-associated changes in BSCFA concentrations in F0 are inherited across generations

In the F0 generation, the Stevia group exhibited significantly lower concentrations of isobutyrate and isovalerate compared to the Control group (*p* = 0.001 and *p* = 0.001, respectively; Figures 6F, G) and lower isobutyrate concentrations compared to the Sucralose group (*p* < 0.01; Figure 6F). Total BSCFA concentrations and proportions were reduced in the Stevia group compared to both the Control and Sucralose groups (*p* = 0.001 and *p* = 0.002; *p* = 0.01 and *p* = 0.002, respectively; Figure 6H, Supplementary Table S8). In F1, both Sucralose and Stevia groups had lower isobutyrate and isovalerate relative to controls group (*p* = 0.017 and *p* = 0.004; *p* = 0.001 and *p* = 0.003, respectively; Figures 6F, G). Additionally, the Sucralose group had higher isobutyrate proportion compared to the Stevia group (*p* = 0.01; Supplementary Table S8). Total BSCFA concentrations were reduced in both groups compared to controls (*p* = 0.02 for Sucralose, *p* = 0.001 for Stevia; Figure 6H), while proportions of total BSCFA remained unchanged compared to controls, except for higher proportions in Sucralose vs. Stevia (*p* = 0.009; Supplementary Table S8). In F2, the Sucralose group had lower isobutyrate concentrations, and the Stevia group lower concentrations and proportions of both BSCFA compared to controls (Sucralose: *p* = 0.006; Stevia: *p* = 0.001 and *p* = 0.005, respectively; Figures 6F, G, Supplementary Table S8). Only the Stevia group showed significant lower concentrations and proportions of total BSCFA compared to controls (*p* = 0.006; Figure 6H, Supplementary Table S8).

The intergenerational analysis by group did not reveal any significant differences in BSCFA concentrations and proportions within the Control and Stevia groups. However, the Sucralose group showed lower isobutyrate concentrations in F1 animals compared to F0 (*p* = 0.04; Figures 6F–H and Supplementary Table S9).

When considering changes across the three generations, the isobutyrate concentrations in the Sucralose group correlated with the abundance of Atopobiaceae and *Olsenella* (*R* = 0.73, *p* = 0.00024 for each one), and propionate concentrations correlated negatively with Patescibacteria (*R* = −0.68, *p* = 0.00015). In the Stevia group, butyrate concentrations correlated with Patescibacteria and Candidatus *Saccaharimonas* (*R* = 0.74, *p* = 5.4 e^−5^ each one), Prevotellaceae (*R* = 0.78, *p* = 1.3 e^−5^), and Ruminococaceae_UCG-010 (*R* = 0.0.73, *p* = 7.4 e^−5^) abundances, while propionate concentrations correlated with *Clostridium*_family_XIII (*R* = 0.76, *p* = 2.9 e^−5^), and isobutyrate and total BSCFA concentrations with Ruminococaceae_UCG-013 (*R* = 0.85, *p* = 2.4 e^−7^, and *R* = 0.83, *p* = 9.5 e^−7^).

## Discussion

The Developmental Origins of Health and Disease (DOHaD) hypothesis proposes that early environmental factors, including maternal stress, nutrition, and infections, shape long-term offspring health and susceptibility to non-communicable diseases (44). Within this framework, the gut microbiota emerges as a key environmental factor, influenced by maternal health, diet, delivery mode, breastfeeding, and early antibiotic exposure, all of which may have lasting health effects (45). The dynamic interplay between diet, microbiota, and epigenetics is particularly critical: diet modulates microbiota composition, which in turn influences epigenetic regulation and metabolic programming. These changes can be transmitted across generations, with enduring consequences for metabolic health. Therefore, our study aimed to evaluate the intergenerational effects of parental consumption of two widely used NNS, sucralose and stevia, on phenotype, hepatic and intestinal gene expression, gut microbiota composition, and fecal SCFA profiles in mice.

### NNS and glucose metabolism

Although parental consumption of sucralose and stevia did not significantly affect glucose metabolism in the F0 generation, notable effects were observed in the offspring, particularly among males, with sucralose-associated alterations persisting into the F2 generation. These findings align with our previous report showing increased weight gain in male F1 and F2 offspring exposed to NNS (31), and with other studies reporting higher adiposity and decreased insulin sensitivity in male offspring following maternal sucralose exposure (26). However, contrasting results, such as lower body weight and glycemia in F1 offspring, have also been described (46). Such discrepancies likely stem from differences in experimental design and whether analyses were sex-disaggregated. Collectively, the evidence suggests that the intergenerational metabolic effects of sucralose are more pronounced in males and persist beyond one generation, whereas those of stevia appear milder and limited to direct offspring.

The different intergenerational effects of sucralose and stevia on glucose metabolism may be explained by their distinct impacts on intestinal glucose sensing and transport. Sucralose stimulates SGLT-1 and GLUT2 expression, enhances activation of sweet taste receptors (T1R2/T1R3), and increases incretin secretion (GLP-1 and GIP), while also inducing TLR4 and impairing insulin signaling pathways (IRS1/AKT) (47). In contrast, stevia elicits weaker activation of these transporters and receptors (47). In addition, as previously stated, sucralose exert prooxidant activities in cells and organs, while stevia is more antioxidant; such difference could also contribute to their differential effect of glucose metabolism. These differences suggest that sucralose exerts a stronger alteration of glucose homeostasis, whereas stevia produces milder effects consistent with the less pronounced metabolic alterations observed in our study.

### Effect of NNS on intestinal and hepatic gene expression

The effect of parental NNS intake on gene expression at the intestinal and liver level was also evaluated. Parental NNS consumption influenced inflammation and metabolism-related gene expression, with sucralose intake increasing intestinal *Tlr4* and *Tnf* expression in the parental and F1 generations, while the effect of stevia only affected the F1 generation. As recently described, these animals also exhibited a greater expression of *Hdac3* in their intestine (31). Although HDAC3 is generally considered as a repressor of *Tlr4* and *Tnf* expression, recent studies suggest that it acts as a dichotomous transcriptional activator and repressor, with a non-canonical deacetylase-independent function that activates the expression of inflammatory genes (48, 49), supporting therefore our results. Although similar effects of sucralose on *Tlr4* and *Tnf* expression have been reported previously in different models (47, 50), this is the first time that this effect is shown to be transmitted to the following generation. TLR4 expressed in intestinal epithelial cells responds to elevated luminal lipopolysaccharides (LPS) by triggering NF-κB-mediated inflammatory pathways, increasing the expression of pro-inflammatory cytokines such as *Tnf* . In this context, it is interesting that *Tlr4* expression correlated with the abundance of gram-negative Patescibacteria, Saccharimonadaceae, and Candidatus_Saccharimonas. Prolonged NNS exposure could therefore act as a potential driver of intestinal inflammation. Similar mechanisms have been described in inflammatory bowel disease, where dysbiosis, increased intestinal permeability, and activation of dendritic cells and macrophages lead to higher expression of TLR4 and other innate immune receptors, resulting in the release of pro-inflammatory cytokines (51). This suggests that NNS exposure may trigger comparable innate and adaptive immune responses, promoting persistent intestinal inflammation. A possible mechanism could be the lesser production of butyrate, a SCFA known for its anti-inflammatory properties. Previous studies have also shown that increased TNF-α expression disrupts tight junction integrity in epithelial cells, thereby compromising barrier function and increasing intestinal permeability (52, 53). Despite elevated *Tnf* levels, no changes in *Tjp1* expression were observed in our study, confirming previous observations that reported that sucralose exposure does not affect intestinal barrier function *in vitro* (or only at high concentrations and long incubation time), and neither does stevia (54, 55). However, ZO-1 function depends not only on transcription but also on its localization and its interaction with other tight-junction proteins including claudins, occludin, tricellulin, JAMs, and polarity complexes (PAR-3/PAR-6/aPKC; Crumbs/PALS1/PATJ). While *Tjp1* expression remained unchanged, our results do not exclude alterations in other tight junction proteins such as claudins or occludins (27), or changes in the positioning and organization of ZO-1 within the tight junction complex.

Regarding the lipogenesis-related gene, *Srebp1*, only parental sucralose decreased its expression in the liver, an effect that persisted in both the F1 and F2 generations. The decrease observed with sucralose might be considered as beneficial, as *Srebp1* stimulates liver lipogenesis and triglyceride accumulation. However, lower hepatic *Srebp1* expression has also been associated with some negative aspects such as persistent hyperglycemia associated with an upregulation of gluconeogenesis gene expression and a decrease in glycolysis and glycogen synthesis gene expression (56). This could explain the changes in glycemia reported above in our animals. Few studies have examined the effect of NNS on *Srebp1* expression and their results contrast with ours. Wu et al. (57) observed that sucralose enhanced *Srebp1* expression and hepatic triglyceride accumulation in mice fed a high-fat diet, and another study reported this NNS (15 mg/ml) increased *Srebp1* expression in white adipose tissue, whereas stevia (25 mg/ml) had no effect (58). Such discrepancies are likely due to differences in the animal model or the concentration of sucralose used. On the other hand, *in vitro* and animal studies have shown that exposure to sucralose increases oxidative stress and inflammation in the liver (22). Since oxidative stress affects SUMOylating enzymes involved in posttranslational protein modification and the regulation of protein function (59, 60), this mechanism could explain our results as well. Globally, our results indicate that parental consumption of sucralose affects the expression of genes involved in the regulation of inflammation and metabolism in the intestine and liver, that these changes are transmitted to the F1 generation but normalize in the F2 generation only in intestine, while the effects of stevia appear only in the F1 generation.

### NNS and microbiota diversity and composition

Another aspect examined in this study was the effect of parental consumption of NNS on gut microbiota and SCFAs. It is estimated that about 85% of the ingested sucralose and more than 95% of the steviosides reach the colon and interact with the microbiota. Sucralose or stevia were shown to alter both the diversity and composition of the microbiota in the mothers and their offspring, confirming results from previous studies (17–19). However, sucralose increased α-diversity in the F1 and F2 generations, while stevia decreased it, relative to F0. Our results with sucralose confirm those of Olivier-Van Stichelen et al. (46), but contrast with those of Dai et al., (27) probably because the offspring in that study were fed a high-fat diet. These contrasting effects may reflect differences in how each sweetener interacts with the colonic microbiota. Sucralose is poorly metabolized and largely excreted in stools. Therefore, it remains unchanged across the colon and could exert greater selective pressure on gut microbiota. In contrast, steviol glycosides are rapidly hydrolyzed by gut bacteria, releasing steviol which is absorbed into the plasma mainly as steviol glucuronide. This process possibly attenuates the impact of stevia on bacterial composition and function. Additionally, sucralose-driven alterations in the maternal microbiota during the F0 generation may influence the establishment and succession of bacterial communities in offspring. Vertical transmission during birth and lactation shapes early colonization, and therefore, greater disruption of the maternal microbiota by sucralose could result in greater alterations in microbial inheritance and consequently, more persistent metabolic effects across generations.

In terms of composition, sucralose caused greater changes in the microbiota than stevia across all generations. This is supported by the fact that taxa from the core microbiota (i.e. consistently present in all subjects), critical for maintaining gut homeostasis and resilience to environmental perturbations, were affected by sucralose, but not by stevia. Among the changes induced by sucralose were the reduction of *Oscillibacter* in the F0 and F1 generations and the increase of *Candidatus*_*Saccharimonas* across F0, F1, and F2 generations. *Oscillibacter* is a butyrate-producing symbiont associated with gut health and improved lipid parameters, which can exhibit potential cardiovascular benefits (61), while *Candidatus_Saccharimonas* belongs to the Erysipelotrichaceae family and has been associated with inflammatory states (62). The effects of sucralose on the gut microbiota therefore appear more robust than those of stevia, as several studies consistently report microbial alterations with sucralose (17–19, 24, 27, 29, 58), whereas the impact of stevia remains less clear. Previous research on stevia has yielded contradictory results: some studies suggest potential benefits due to prebiotic-like properties (24, 55), while others describe effects similar to those observed with other NNS (47, 51, 58).

Changes were also detected in less common taxa (i.e. from the non-core microbiota) that may also have significant health effects and were passed on to offspring. Sucralose, again, had a more pronounced effect than stevia, including increased abundance of *Streptococcus* and *Ureaplasma*. These changes could increase the risk of inflammatory and metabolic diseases in the offspring of individuals exposed to sucralose. In the Sucralose and Stevia groups, *Alloprevotella* and *Lactobacillus* were higher in the F1 generation and *Turicibacter* in the F2. *Lactobacillus, Alloprevotella*, and *Turicibacter* have contradictory roles in metabolic health (19, 63, 64). Some species of *Lactobacillus* have been associated with both obesity and metabolic diseases, while other species are found in lower abundance in these diseases, or are used as beneficial probiotics (65). Regarding *Turicibacter*, this genus has been linked to positive effects on metabolic health (66, 67).

These shifts suggest disrupted microbiota transmission between mothers and offspring, as indicated by β-diversity variations. LEfSe analysis shows sucralose-associated enrichment of *Candidatus Stoquefichus, Lactococcus*, and *Coprococcus_3* in F0, *Roseburia* enrichment in F1, and Patescibacteria, Saccharimonadaceae, *Candidatus_Saccharimonas*, and *Anaerostipes* in F2, while stevia showed no specific enrichment. Although *Coprococcus_3, Roseburia*, and *Anaerostipes* are butyrate-producing genera, generally associated to improved inflammation and metabolism, their increased abundance was not associated with higher levels of fecal butyrate in our study, these concentrations in the Sucralose group being similar or even lower than in the Control group in all the generations. In addition, sucralose also increased the pro-inflammatory *Desulfovibrio*. Similar microbiota alterations have been observed in murine models of lupus, suggesting a potential link to immune dysregulation (62).

Overall, these results suggest that sucralose consumption is associated with a greater effect on the microbiota composition than stevia, and that some of these changes are passed on to the subsequent generations.

### NNS and SCFA/BSCFA levels

Fecal SCFA concentrations depend on dietary fiber availability, the equilibrium between SCFA-producing and SCFA-consuming bacteria, and SCFA colonic absorption via MCT1 and SMCT1 transporters (30, 68, 69). Our study shows lower fecal SCFA concentrations in the F0 animals supplemented with sucralose or stevia, with acetate and valerate being the most affected. As fiber intake was similar in the three groups and there is no evidence that NNS modulate MCT1 and SMCT1 expression in the colonic epithelium, these reductions are likely due to changes in microbial composition. Furthermore, this phenomenon was observed despite the presence of pro-inflammatory signals (increased TNF expression), which are known to downregulate MCT1 (69). Although some butyrate-producing taxa were enriched in sucralose-treated animals, their low abundance suggests that they probably do not contribute significantly to the fecal butyrate pool. Notably, SCFA reduction persisted in the F1 and F2 generations, with further declines in propionate and butyrate, and no significant differences between the two NNS groups. These findings are consistent with previous animal studies, which reported reduced cecal butyrate and trends toward lower acetate and propionate in offspring of sucralose-exposed mice (27). Interestingly, we previously reported a higher expression of *Hdac3* in the intestine of mice consuming sucralose and their offspring (31). Such findings could be therefore explained by the lower concentrations of fecal propionate and butyrate, considered as potent inhibitors of *Hdac3*. On the other hand, diminutions in the concentrations of isobutyrate and isovalerate, produced from branched-chain amino acid fermentation, were also observed in stevia-treated F0 animals. In the F1 and F2 generations, reduced BSCFA levels were observed in both NNS groups, suggesting diminished protein fermentation and potentially lower production of harmful byproducts such as ammonia, hydrogen sulfide, phenol, and p-cresol (70). These microbial metabolite shifts may influence inflammatory signaling and gene expression, particularly given the association between low SCFA levels and increased risk of obesity and chronic intestinal inflammation (71). Collectively, our results indicate that NNS consumption can disrupt microbial fermentation activity across generations. The intergenerational persistence of SCFA and BSCFA alterations highlights the need for further investigation into their long-term physiological impact.

It is possible that alterations in the F0 microbiota, along with inadequate microbial transmission from mothers to offspring, contributed to the observed changes in SCFA concentrations and gene expression. Butyrate is anti-inflammatory and a well-known HDAC3 inhibitor. Therefore, a lower production of this SCFA probably contributes to the increased levels of inflammatory biomarkers directly and indirectly through increased *Hdac3* expression (31), histone deacetylation, and suppression of the expression of genes involved in metabolic regulation and immune responses. These findings suggest that alterations in parental microbiota may play a significant role in the intergenerational effects of NNS consumption, potentially influencing metabolic health and gene expression across generations.

### Limitations

The experimental design does not allow us to disentangle the intergenerational effects of perinatal exposure from those of gestational exposure. Because both parents may exert significant influences on offspring metabolic health, future studies should aim to isolate and evaluate the contribution of each progenitor independently. Such approaches would improve our understanding of the mechanisms underlying the intergenerational effects of NNS.

In this study, we focused on fecal microbiota rather than cecal microbiota because fecal samples more accurately reflect the microbial communities transmitted from the dam to the offspring through maternal contact, environmental exposure, and early-life interactions. In contrast, cecal microbiota are not directly transferred to pups, making fecal sampling more appropriate for studying microbiota-mediated intergenerational effects.

Although the use of multiple reference genes is increasingly recommended, we used a single validated housekeeping gene, which remains acceptable when its stability is empirically confirmed within the specific tissues and experimental conditions under investigation.

## Conclusion

In summary, our findings demonstrate that parental consumption of sucralose or stevia induces persistent, intergenerational changes in host metabolism, intestinal and hepatic gene expression, gut microbiota composition, and microbial metabolite production in unexposed offspring. These results challenge the long-standing assumption that non-nutritive sweeteners are metabolically inert and underscore their potential to influence offspring health through microbial and molecular pathways. Given the widespread use of NNS during critical developmental periods, these findings raise important questions about their safety and long-term impact. Future research should aim to clarify the mechanisms underlying these intergenerational effects, their reversibility, and relevance to human health. A deeper understanding of how NNS shape host–microbiota interactions across generations is essential for developing evidence-based dietary guidelines and informing public health policies.

## Data Availability

The data presented in this study are publicly available. The data can be found here: https://www.ncbi.nlm.nih.gov/sra, accession PRJNA1285496.
