# Supplementary material for: Artificial and natural non-nutritive sweeteners drive divergent gut and genetic responses across generations
Source: Front Nutr. 2026 Apr 10;13:1694149. doi: 10.3389/fnut.2026.1694149 (PMC13106605; doi:10.3389/fnut.2026.1694149)
Supplement: Supplementary file 1 [file Supplementary_file_1.docx]

**Supplementary Tables**

**Table S1.** Sequences of the different primers used in the study

| **Gene** | **Sequence** |
| --- | --- |
| **V3-v4 region** (33)  341Forward (5’-3’)  785Reverse (5’-3’) | CCTACGGGNGGCWGCAG  GACTACHVGGGTATCTAATCC |
| **β-actin** (38)  Forward (5’-3’)  Reverse (5’-3’) | CCTTCTTGGGTATGGAATCCTGT  CACTGTGTTGGCATAGAGGTCTTTAC |
| **Srebp1** (39)  Forward (5’-3’)  Reverse (5’-3’) | GTGAGCCTGACAAGCAATCA  GGTGCCTACAGAGCAAGAGG |
| **Tlr4** (40)  Forward (5’-3’)  Reverse (5’-3’) | GCCTTTCAGGGAATTAAGCTCC  AGATCAACCGATGGACGTGTAA |
| **Tnf** (41)  Forward (5’-3’)  Reverse (5’-3’) | TGGGACAGTGACCTGGACTGT  TTCGGAAAGCCCATTTGAGT |
| **Tjp1** (42)  Forward (5’-3’)  Reverse (5’-3’) | GCCGCTAAGAGCACAGCAA  TCCCCACTCTGAAAATGAGGA |

**Table S2.** Description of the core microbiota at the genus level (i.e. the genera present in 95% of all animals)

| **Genera forming the core microbiota** |
| --- |
| **Bacteroidetes phylum**  *Bacteroides*, *Alistipes*, *Parabacteroides*, *Odoribacter*, *Rikenella*, *Muribaculum*, unidentified genera of the Muribaculaceae family, Prevotellaceae UCG-001, Rikenellaceae RC9 gut group, Prevotellaceae Ga6A1 group.  **Firmicutes phylum**  *Oscillibacter*, *Intestinimonas*, *Ruminococcus* 1, *Ruminiclostridium*, *Ruminiclostridium* 5, *Ruminiclostridium* 9, *Tyzzerella*, *Tyzzerella* 3, *Lachnoclostridium*, unidentified genera of the Clostridiales vadin BB60 group, Ruminococcaceae, and Lachnospiraceae, Lachnospiraceae UCG-006, Lachnospiraceae UCG-001, Lachnospiraceae NK4A136 group, GCA-900066575, Ruminococcaceae UCG-010, Ruminococcaceae NK4A214 group, Ruminococcaceae UCG-014, Christensenellaceae R-7 group.  **Patescibacteria phylum**  Candidatus *Saccharimonas*.  **Proteobacteria phylum**  *Helicobacter*.  **Actinobacteria phylum**  *Bifidobacterium,* *Enterorhabdus*  **Cyanobacteria phylum**  Unidentified genera of the Gastranaerophilales order. |

**Table S3.** Significant intergroup differences in the composition of the fecal microbiota of the parental generation (F0)

|  |  | **Relative abundance of bacterial taxa in generation F0** | | | | | | | | | | |
| --- | --- | --- | --- | --- | --- | --- | --- | --- | --- | --- | --- | --- |
|  | | Control (n=6) | | | Sucralose (n=6) | | | | Stevia (n=6) | | | |
| Phylum | Patescibacteria | 0,0247 | + | 0,0162_ab_ | | 0,0302 | + | 0,0105_a_ | | 0,0177 | + | 0,0063_b_ |
| Genus | |  | | |  | | | |  | | | |
| Actinobacteria | *Olsenella* | 0,0009 | + | 0,0007_ab_ | | 0,0031 | + | 0,0045_a_ | | 0,0002 | + | 0,0005_b_ |
| Bacteroidetes | *Parabacteroides* | 0,0099 | + | 0,0083_a_ | | 0,0062 | + | 0,0030_b_ | | 0,0096 | + | 0,0028_a_ |
| Firmicutes | *Jeotgalicoccus* | 0,0000 | + | 0,0000_a_ | | 0,0001 | + | 0,0001_b_ | | 0,0000 | + | 0,0000_a_ |
|  | *Staphylococcus* | 0,0000 | + | 0,0000_a_ | | 0,0006 | + | 0,0006_b_ | | 0,0001 | + | 0,0003_ab_ |
|  | *Lactobacillus* | 0,0000 | + | 0,0232_a_ | | 0,0676 | + | 0,0589_b_ | | 0,0477 | + | 0,0137_ab_ |
|  | *Lactococcus* | 0,0000 | + | 0,0000_a_ | | 0,0011 | + | 0,0015_b_ | | 0,0001 | + | 0,0002_a_ |
|  | *Streptococcus* | 0,0000 | + | 0,0000_a_ | | 0,0001 | + | 0,0000_b_ | | 0,0000 | + | 0,0000_ab_ |
|  | *Candidatus Arthromitus* | 0,0008 | + | 0,0017_ab_ | | 0,0046 | + | 0,0020_a_ | | 0,0002 | + | 0,0007_b_ |
|  | *Clostridium sensu stricto 1* | 0,0055 | + | 0,0363_a_ | | 0,0000 | + | 0,0001_a_ | | 0,0251 | + | 0,0098_b_ |
|  | *Coprococcus 3* | 0,0010 | + | 0,0008_a_ | | 0,0017 | + | 0,0008_b_ | | 0,0009 | + | 0,0003_a_ |
|  | *Acetatifactor* | 0,0004 | + | 0,0005_a_ | | 0,0005 | + | 0,0001_ab_ | | 0,0012 | + | 0,0011_b_ |
|  | *Lachnospiraceae FCS020 group* | 0,0024 | + | 0,0016_a_ | | 0,0003 | + | 0,0002_b_ | | 0,0025 | + | 0,0016_a_ |
|  | *Romboutsia* | 0,0068 | + | 0,0118_a_ | | 0,0000 | + | 0,0000_b_ | | 0,0015 | + | 0,0043_ab_ |
|  | *Harryflintia* | 0,0000 | + | 0,0001_a_ | | 0,0002 | + | 0,0001_ab_ | | 0,0003 | + | 0,0002_b_ |
|  | *Oscillibacter* | 0,0106 | + | 0,0039_a_ | | 0,0042 | + | 0,0010_b_ | | 0,0088 | + | 0,0025_ab_ |
|  | *Ruminiclostridium 9* | 0,0157 | + | 0,0086_a_ | | 0,0084 | + | 0,0023_b_ | | 0,0150 | + | 0,0009_a_ |
|  | *Ruminococcaceae UCG-013* | 0,0003 | + | 0,0003_ab_ | | 0,0004 | + | 0,0003_a_ | | 0,0001 | + | 0,0001_b_ |
|  | *Ruminococcaceae UCG-014* | 0,0363 | + | 0,0438_a_ | | 0,0148 | + | 0,0077_b_ | | 0,0194 | + | 0,0037_ab_ |
|  | *Candidatus Stoquefichus* | 0,0000 | + | 0,0000_a_ | | 0,0020 | + | 0,0019_b_ | | 0,0002 | + | 0,0001_a_ |
|  | *Dubosiella* | 0,0000 | + | 0,0132_ab_ | | 0,0000 | + | 0,0000_a_ | | 0,0346 | + | 0,0311_b_ |
|  | *Erysipelatoclostridium* | 0,0000 | + | 0,0002_a_ | | 0,0004 | + | 0,0005_ab_ | | 0,0009 | + | 0,0014_b_ |
|  | *Turicibacter* | 0,0007 | + | 0,0036_ab_ | | 0,0000 | + | 0,0001_a_ | | 0,0257 | + | 0,0264_b_ |
| Patescibacteria | *Candidatus Saccharimonas* | 0,0247 | + | 0,0162_ab_ | | 0,0302 | + | 0,0105_a_ | | 0,0177 | + | 0,0063_b_ |
| Proteobacteria | *Bilophila* | 0,0000 | + | 0,0001_a_ | | 0,0003 | + | 0,0003_b_ | | 0,0002 | + | 0,0002_ab_ |
|  | *Desulfovibrio* | 0,0000 | + | 0,0021_a_ | | 0,0056 | + | 0,0021_b_ | | 0,0027 | + | 0,0033_a_ |
|  | *Acinetobacter* | 0,0000 | + | 0,0000_a_ | | 0,0003 | + | 0,0008_b_ | | 0,0000 | + | 0,0000_a_ |
| Tenericutes | *Ureaplasma* | 0,0000 | + | 0,0002_a_ | | 0,0010 | + | 0,0011_b_ | | 0,0002 | + | 0,0005_ab_ |

Values represent median ± IQR. Kruskal-Wallis test, multiple comparisons using Dunn's test adjusted with Bonferroni correction, different letters indicate differences at p < 0.05.

|  |  | **Relative abundance of bacterial taxa in generation F1** | | | | | | | | |  |
| --- | --- | --- | --- | --- | --- | --- | --- | --- | --- | --- | --- |
|  | | Control (n=6) | | | Sucralose (n=6) | | | Stevia (n=6) | | | |
| Phylum | Deferribacteres | 0,0000 | + | 0,0001_a_ | 0,0008 | + | 0,0012_ab_ | 0,0028 | + | 0,0016_b_ | |
|  | Patescibacteria | 0,0133 | + | 0,0048_a_ | 0,0416 | + | 0,0271_b_ | 0,0155 | + | 0,0071_ab_ | |
|  | Cyanobacteria | 0,0113 | + | 0,0092_a_ | 0,0011 | + | 0,0011_b_ | 0,0039 | + | 0,0026_a_ | |
| Genus | |  | | |  | | |  | | | |
| Actinobacteria | *Bifidobacterium* | 0,0258 | + | 0,0308_a_ | 0,0027 | + | 0,0041_b_ | 0,0059 | + | 0,0034_ab_ | |
|  | *DNF00809* | 0,0012 | + | 0,0018_ab_ | 0,0023 | + | 0,0015_a_ | 0,0007 | + | 0,0008_b_ | |
|  | *Enterorhabdus* | 0,0017 | + | 0,0015_a_ | 0,0042 | + | 0,0015_b_ | 0,0026 | + | 0,0026_ab_ | |
| Bacteroidetes | *Odoribacter* | 0,0059 | + | 0,0043_ab_ | 0,0118 | + | 0,0105_a_ | 0,0037 | + | 0,0025_b_ | |
|  | *Alloprevotella* | 0,0000 | + | 0,0001_a_ | 0,0411 | + | 0,0287_b_ | 0,0419 | + | 0,0301_b_ | |
|  | *Prevotellaceae Ga6A1_group* | 0,0113 | + | 0,0219_a_ | 0,0021 | + | 0,0019_b_ | 0,0111 | + | 0,0057_a_ | |
|  | *Prevotellaceae UCG-001* | 0,0527 | + | 0,0161_a_ | 0,0297 | + | 0,0072_b_ | 0,0460 | + | 0,0156_ab_ | |
|  | *Rikenella* | 0,0018 | + | 0,0013_ab_ | 0,0032 | + | 0,0026_a_ | 0,0007 | + | 0,0008_b_ | |
| Deferribacteres | *Mucispirillum* | 0,0000 | + | 0,0001_a_ | 0,0008 | + | 0,0012_ab_ | 0,0028 | + | 0,0016_b_ | |
| Firmicutes | *Lactobacillus* | 0,0000 | + | 0,0001_a_ | 0,0929 | + | 0,1220_b_ | 0,0854 | + | 0,0562_a_ | |
|  | *Streptococcus* | 0,0000 | + | 0,0000_a_ | 0,0001 | + | 0,0005_b_ | 0,0000 | + | 0,0000_a_ | |
|  | *Candidatus Arthromitus* | 0,0004 | + | 0,0010_ab_ | 0,0025 | + | 0,0038_a_ | 0,0001 | + | 0,0002_b_ | |
|  | *Clostridium sensu stricto 1* | 0,0226 | + | 0,0199_a_ | 0,0106 | + | 0,0238_a_ | 0,0700 | + | 0,0368_b_ | |
|  | *Lachnospiraceae NK4A136 group* | 0,1704 | + | 0,0537_a_ | 0,1073 | + | 0,0624_ab_ | 0,0906 | + | 0,0452_b_ | |
|  | *Roseburia* | 0,0014 | + | 0,0052_a_ | 0,0157 | + | 0,0067_b_ | 0,0011 | + | 0,0027_b_ | |
|  | *Peptococcus* | 0,0000 | + | 0,0000_a_ | 0,0001 | + | 0,0003_b_ | 0,0000 | + | 0,0002_ab_ | |
|  | *Anaerotruncus* | 0,0004 | + | 0,0006_ab_ | 0,0016 | + | 0,0020_a_ | 0,0000 | + | 0,0002_b_ | |
|  | *Candidatus Soleaferrea* | 0,0009 | + | 0,0004_a_ | 0,0006 | + | 0,0004_a_ | 0,0002 | + | 0,0003_b_ | |
|  | *GCA-900066225* | 0,0000 | + | 0,0001_ab_ | 0,0002 | + | 0,0002_a_ | 0,0000 | + | 0,0000_b_ | |
|  | *Intestinimonas* | 0,0035 | + | 0,0040_ab_ | 0,0085 | + | 0,0050_a_ | 0,0034 | + | 0,0022_b_ | |
|  | *Oscillibacter* | 0,0143 | + | 0,0069_a_ | 0,0047 | + | 0,0027_b_ | 0,0069 | + | 0,0048_ab_ | |
|  | *Candidatus Stoquefichus* | 0,0000 | + | 0,0000_a_ | 0,0003 | + | 0,0012_b_ | 0,0002 | + | 0,0003_ab_ | |
|  | *Dubosiella* | 0,0000 | + | 0,0001_a_ | 0,0000 | + | 0,0013_a_ | 0,0173 | + | 0,0282_b_ | |
|  | *Erysipelatoclostridium* | 0,0000 | + | 0,0000_a_ | 0,0017 | + | 0,0029_b_ | 0,0000 | + | 0,0003_ab_ | |
| Patescibacteria | *Candidatus Saccharimonas* | 0,0133 | + | 0,0048_a_ | 0,0416 | + | 0,0271_b_ | 0,0155 | + | 0,0071_ab_ | |
| Proteobacteria | *Desulfovibrio* | 0,0000 | + | 0,0000_a_ | 0,0036 | + | 0,0021_b_ | 0,0026 | + | 0,0035_ab_ | |
|  | *Parasutterella* | 0,0000 | + | 0,0001_a_ | 0,0051 | + | 0,0042_ab_ | 0,0058 | + | 0,0052_b_ | |
| Tenericutes | *Ureaplasma* | 0,0000 | + | 0,0000_a_ | 0,0001 | + | 0,0001_b_ | 0,0000 | + | 0,0002_ab_ | |

**Table S4.** Significant intergroup differences in the composition of the fecal microbiota of the F1 generation.

Values represent median ± IQR. Kruskal-Wallis test, multiple comparisons using Dunn's test adjusted with Bonferroni correction, different letters indicate differences at p < 0.05.

**Table S5.** Significant intergroup differences in the composition of the fecal microbiota of the F2 generation.

|  |  | **Relative abundance of bacterial taxa in generation F2** | | | | | | | | | |
| --- | --- | --- | --- | --- | --- | --- | --- | --- | --- | --- | --- |
|  | | Control (n=6) | | | Sucralose (n=6) | | | Stevia (n=6) | | | |
| Phylum | Bacteroidetes | 0,5169 | + | 0,0487_a_ | 0,4270 | + | 0,0519_b_ | 0,4765 | | + | 0,0578_ab_ |
|  | Cyanobacteria | 0,0034 | + | 0,0032_a_ | 0,0023 | + | 0,0036_ab_ | 0,0011 | | + | 0,0008_b_ |
|  | Tenericutes | 0,0012 | + | 0,0019_ab_ | 0,0040 | + | 0,0023_a_ | 0,0005 | | + | 0,0006_b_ |
|  | Patescibacteria | 0,0068 | + | 0,0057_a_ | 0,0276 | + | 0,0095_b_ | 0,0129 | | + | 0,0036_a_ |
| Genus | |  | | |  | | |  | | | |
| Bacteroidetes | *Muribaculum* | 0,0161 | + | 0,0034_a_ | 0,0103 | + | 0,0026_b_ | | 0,0117 | + | 0,0041_ab_ |
|  | *Rikenella* | 0,0019 | + | 0,0014_a_ | 0,0022 | + | 0,0010_a_ | | 0,0006 | + | 0,0010_b_ |
| Firmicutes | *Streptococcus* | 0,0000 | + | 0,0000_a_ | 0,0001 | + | 0,0001_b_ | 0,0000 | | + | 0,0000_a_ |
|  | *Clostridium sensu stricto 1* | 0,0310 | + | 0,0351_a_ | 0,0321 | + | 0,0223_a_ | 0,0069 | | + | 0,0073_b_ |
|  | *Anaerostipes* | 0,0000 | + | 0,0000_a_ | 0,0018 | + | 0,0012_b_ | 0,0000 | | + | 0,0000_a_ |
|  | *Lachnospiraceae UCG-006* | 0,0149 | + | 0,0031_a_ | 0,0019 | + | 0,0018_b_ | 0,0184 | | + | 0,0155_a_ |
|  | *Roseburia* | 0,0028 | + | 0,0017_a_ | 0,0067 | + | 0,0076_b_ | 0,0022 | | + | 0,0028_a_ |
|  | *Butyricicoccus* | 0,0013 | + | 0,0009_a_ | 0,0033 | + | 0,0014_b_ | 0,0020 | | + | 0,0022_ab_ |
|  | *GCA-900066225* | 0,0000 | + | 0,0001_a_ | 0,0002 | + | 0,0001_b_ | 0,0000 | | + | 0,0000_a_ |
|  | *Intestinimonas* | 0,0038 | + | 0,0032_a_ | 0,0083 | + | 0,0026_b_ | 0,0053 | | + | 0,0021_ab_ |
|  | *Ruminococcaceae UCG-010* | 0,0009 | + | 0,0006_ab_ | 0,0012 | + | 0,0002_a_ | 0,0006 | | + | 0,0003_b_ |
|  | *Ruminococcus 1* | 0,0116 | + | 0,0070_a_ | 0,0050 | + | 0,0022_a_ | 0,0005 | | + | 0,0015_b_ |
|  | *Turicibacter* | 0,0000 | + | 0,0000_a_ | 0,0090 | + | 0,0144_b_ | 0,0143 | | + | 0,0437_b_ |
| Patescibacteria | *Candidatus Saccharimonas* | 0,0068 | + | 0,0057_a_ | 0,0276 | + | 0,0095_b_ | 0,0129 | | + | 0,0036_a_ |
| Tenericutes | *Ureaplasma* | 0,0001 | + | 0,0004_a_ | 0,0008 | + | 0,0003_b_ | 0,0000 | | + | 0,0002_a_ |

Values represent median ± IQR. Kruskal-Wallis test, multiple comparisons using Dunn's test adjusted with Bonferroni correction, different letters indicate differences at p < 0.05.

**Table S6**. Significant intergenerational differences in the composition of the fecal microbiota of mice in the Sucralose group.

|  |  | **Relative abundance of bacterial taxa in Sucralose groups** | | | | | | | | |
| --- | --- | --- | --- | --- | --- | --- | --- | --- | --- | --- |
|  | | F0 (n=6) | | | F1(n=6) | | | F2 (n=6) | | |
| Phylum | Bacteroidetes | 0,5043 | + | 0,0465_a_ | 0,3910 | + | 0,0325_b_ | 0,4270 | + | 0,0519_ab_ |
|  | Firmicutes | 0,3828 | + | 0,0581_a_ | 0,5229 | + | 0,0493_b_ | 0,4674 | + | 0,0618_ab_ |
|  | Deferribacteres | 0,0022 | + | 0,0021_ab_ | 0,0008 | + | 0,0012_a_ | 0,0032 | + | 0,0031_b_ |
|  | Proteobacteria | 0,0208 | + | 0,0094_ab_ | 0,0129 | + | 0,0061_a_ | 0,0202 | + | 0,0077_b_ |
| Genus | |  | | |  | | |  | | |
| Actinobacteria | *Bifidobacterium* | 0,0130 | + | 0,0129_a_ | 0,0027 | + | 0,0041_b_ | 0,0064 | + | 0,0073_ab_ |
|  | *Olsenella* | 0,0031 | + | 0,0045_a_ | 0,0002 | + | 0,0006_b_ | 0,0003 | + | 0,0022_ab_ |
|  | *DNF00809* | 0,0012 | + | 0,0007_ab_ | 0,0023 | + | 0,0015_a_ | 0,0008 | + | 0,0001_b_ |
|  | *Enterorhabdus* | 0,0029 | + | 0,0005_ab_ | 0,0042 | + | 0,0015_a_ | 0,0020 | + | 0,0008_b_ |
| Bacteroidetes | *Odoribacter* | 0,0072 | + | 0,0023_ab_ | 0,0118 | + | 0,0105_a_ | 0,0035 | + | 0,0021_b_ |
|  | *Prevotellaceae Ga6A1 group* | 0,0122 | + | 0,0158_a_ | 0,0021 | + | 0,0019_b_ | 0,0052 | + | 0,0104_ab_ |
|  | *Prevotellaceae UCG-001* | 0,0494 | + | 0,0120_a_ | 0,0297 | + | 0,0072_b_ | 0,0420 | + | 0,0108_ab_ |
|  | *Parabacteroides* | 0,0062 | + | 0,0030_a_ | 0,0054 | + | 0,0032_a_ | 0,0101 | + | 0,0012_b_ |
| Deferribacteres | *Mucispirillum* | 0,0022 | + | 0,0021_ab_ | 0,0008 | + | 0,0012_a_ | 0,0032 | + | 0,0031_b_ |
| Firmicutes | *Jeotgalicoccus* | 0,0001 | + | 0,0001_a_ | 0,0000 | + | 0,0000_b_ | 0,0000 | + | 0,0000_b_ |
|  | *Staphylococcus* | 0,0006 | + | 0,0006_a_ | 0,0000 | + | 0,0000_b_ | 0,0000 | + | 0,0001_b_ |
|  | *Candidatus_Arthromitus* | 0,0046 | + | 0,0020_a_ | 0,0025 | + | 0,0038_ab_ | 0,0014 | + | 0,0016_b_ |
|  | *Clostridium sensu stricto 1* | 0,0000 | + | 0,0001_a_ | 0,0106 | + | 0,0238_b_ | 0,0321 | + | 0,0223_b_ |
|  | *Acetatifactor* | 0,0005 | + | 0,0001_ab_ | 0,0002 | + | 0,0005_a_ | 0,0016 | + | 0,0020_b_ |
|  | *Coprococcus 3* | 0,0003 | + | 0,0001_a_ | 0,0000 | + | 0,0000_b_ | 0,0000 | + | 0,0000_b_ |
|  | *Lachnospiraceae FCS020 group* | 0,0003 | + | 0,0002_a_ | 0,0015 | + | 0,0007_b_ | 0,0012 | + | 0,0006_b_ |
|  | *Lachnospiraceae UCG-006* | 0,0104 | + | 0,0006_a_ | 0,0057 | + | 0,0041_ab_ | 0,0019 | + | 0,0018_b_ |
|  | *Roseburia* | 0,0047 | + | 0,0066_a_ | 0,0157 | + | 0,0067_b_ | 0,0067 | + | 0,0076_ab_ |
|  | *Tyzzerella* | 0,0009 | + | 0,0004_a_ | 0,0004 | + | 0,0002_b_ | 0,0007 | + | 0,0002_ab_ |
|  | *Romboutsia* | 0,0000 | + | 0,0000_a_ | 0,0149 | + | 0,0167_b_ | 0,0047 | + | 0,0008_b_ |
|  | *Butyricicoccus* | 0,0026 | + | 0,0014_ab_ | 0,0018 | + | 0,0018_a_ | 0,0033 | + | 0,0014_b_ |
|  | *Oscillibacter* | 0,0042 | + | 0,0010_a_ | 0,0047 | + | 0,0027_a_ | 0,0088 | + | 0,0017_b_ |
|  | *Ruminococcaceae NK4A214 group* | 0,0005 | + | 0,0003_a_ | 0,0015 | + | 0,0011_b_ | 0,0009 | + | 0,0002_ab_ |
|  | *Candidatus Stoquefichus* | 0,0020 | + | 0,0019_a_ | 0,0003 | + | 0,0012_b_ | 0,0005 | + | 0,0003_ab_ |
|  | *Turicibacter* | 0,0000 | + | 0,0001_a_ | 0,0037 | + | 0,0193_ab_ | 0,0090 | + | 0,0144_b_ |
| Proteobacteria | *Bilophila* | 0,0003 | + | 0,0003_a_ | 0,0001 | + | 0,0001_b_ | 0,0002 | + | 0,0001_ab_ |
|  | *Desulfovibrio* | 0,0056 | + | 0,0021_a_ | 0,0036 | + | 0,0021_b_ | 0,0021 | + | 0,0018_b_ |
|  | *Parasutterella* | 0,0096 | + | 0,0039_a_ | 0,0051 | + | 0,0042_b_ | 0,0066 | + | 0,0011_ab_ |
|  | *Acinetobacter* | 0,0003 | + | 0,0008_a_ | 0,0000 | + | 0,0000_b_ | 0,0000 | + | 0,0000_b_ |
| Tenericutes | *Ureaplasma* | 0,0010 | + | 0,0011_a_ | 0,0001 | + | 0,0001_b_ | 0,0008 | + | 0,0003_a_ |

Values represent median ± IQR. Kruskal-Wallis test, multiple comparisons using Dunn's test adjusted with Bonferroni correction, different letters indicate differences at p < 0.05.

**Table S7.** Significant intergenerational differences in the composition of the fecal microbiota of mice in the Stevia group.

|  |  | **Relative abundance of bacterial taxa in Stevia groups** | | | | | | | | |
| --- | --- | --- | --- | --- | --- | --- | --- | --- | --- | --- |
|  | | F0 (n=6) | | | F1 (n=6) | | | F2 (n=6) | | |
| Phylum | Cyanobacteria | 0,0053 | + | 0,0044_a_ | 0,0039 | + | 0,0026_a_ | 0,0011 | + | 0,0008_b_ |
|  | Tenericutes | 0,0022 | + | 0,0009_a_ | 0,0023 | + | 0,0019_a_ | 0,0005 | + | 0,0006_b_ |
| Genus | |  | | |  | | |  | | |
| Bacteroidetes | *Rikenella* | 0,0019 | + | 0,0011_a_ | 0,0007 | + | 0,0008_b_ | 0,0006 | + | 0,0010_b_ |
| Firmicutes | *Staphylococcus* | 0,0001 | + | 0,0003_a_ | 0,0000 | + | 0,0000_b_ | 0,0000 | + | 0,0000_ab_ |
|  | *Clostridium sensu stricto 1* | 0,0251 | + | 0,0098_a_ | 0,0700 | + | 0,0368_b_ | 0,0069 | + | 0,0073_a_ |
|  | *Anaerovorax* | 0,0001 | + | 0,0001_a_ | 0,0000 | + | 0,0000_b_ | 0,0000 | + | 0,0000_b_ |
|  | *Lachnospiraceae UCG-006* | 0,0057 | + | 0,0027_a_ | 0,0111 | + | 0,0180_ab_ | 0,0184 | + | 0,0155_b_ |
|  | *Romboutsia* | 0,0015 | + | 0,0043_a_ | 0,0114 | + | 0,0050_b_ | 0,0043 | + | 0,0046_a_ |
|  | *Anaerotruncus* | 0,0012 | + | 0,0005_a_ | 0,0000 | + | 0,0002_b_ | 0,0003 | + | 0,0006_b_ |
|  | *Candidatus Soleaferrea* | 0,0007 | + | 0,0007_a_ | 0,0002 | + | 0,0003_b_ | 0,0005 | + | 0,0004_ab_ |
|  | *GCA-900066225* | 0,0001 | + | 0,0001_a_ | 0,0000 | + | 0,0000_b_ | 0,0000 | + | 0,0000_b_ |
|  | *Harryflintia* | 0,0003 | + | 0,0002_a_ | 0,0000 | + | 0,0001_b_ | 0,0001 | + | 0,0003_ab_ |
|  | *Ruminiclostridium* | 0,0038 | + | 0,0015_a_ | 0,0021 | + | 0,0012_b_ | 0,0021 | + | 0,0010_b_ |
|  | *Ruminococcaceae UCG-005* | 0,0012 | + | 0,0008_a_ | 0,0000 | + | 0,0003_b_ | 0,0000 | + | 0,0003_b_ |
|  | *Ruminococcaceae UCG-010* | 0,0009 | + | 0,0001_ab_ | 0,0010 | + | 0,0006_a_ | 0,0006 | + | 0,0003_b_ |
|  | *Ruminococcaceae UCG-014* | 0,0194 | + | 0,0037_a_ | 0,0185 | + | 0,0121_ab_ | 0,0096 | + | 0,0066_b_ |
|  | *Ruminococcus 1* | 0,0108 | + | 0,0088_a_ | 0,0088 | + | 0,0046_a_ | 0,0005 | + | 0,0015_b_ |

Values represent median ± IQR. Kruskal-Wallis test, multiple comparisons using Dunn's test adjusted with Bonferroni correction, different letters indicate differences at p < 0.05.

**Table S8.** Fecal concentrations of SCFA and BCFA by generation in mice from the Control, Sucralose and Stevia group.


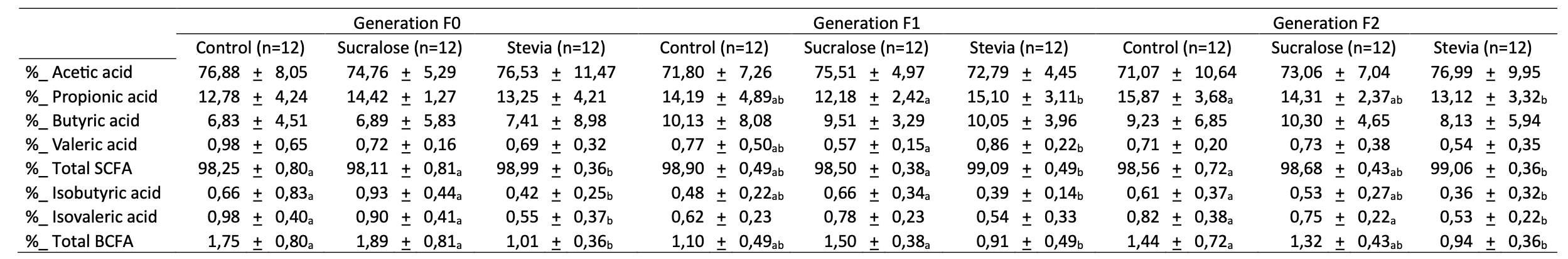


**Table S9.** Fecal concentrations of SCFA and BCFA by treatment groups in mice from theF0, F1 and F2 generation.
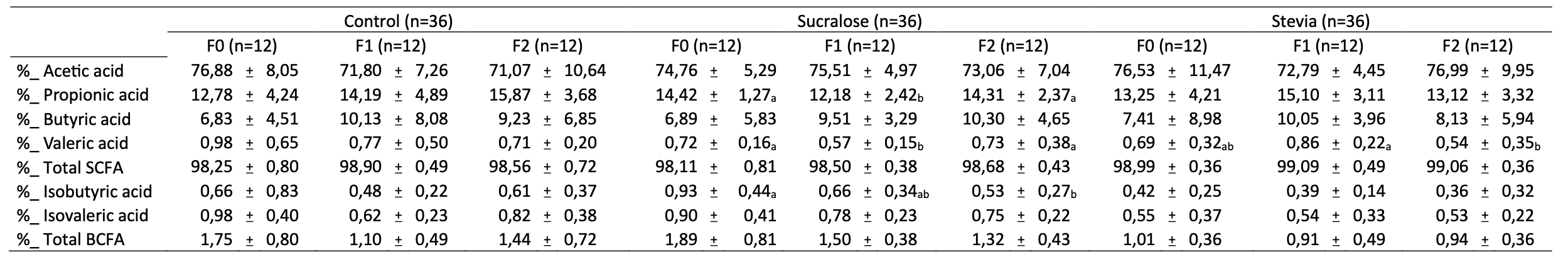


**Supplementary Figures**

**
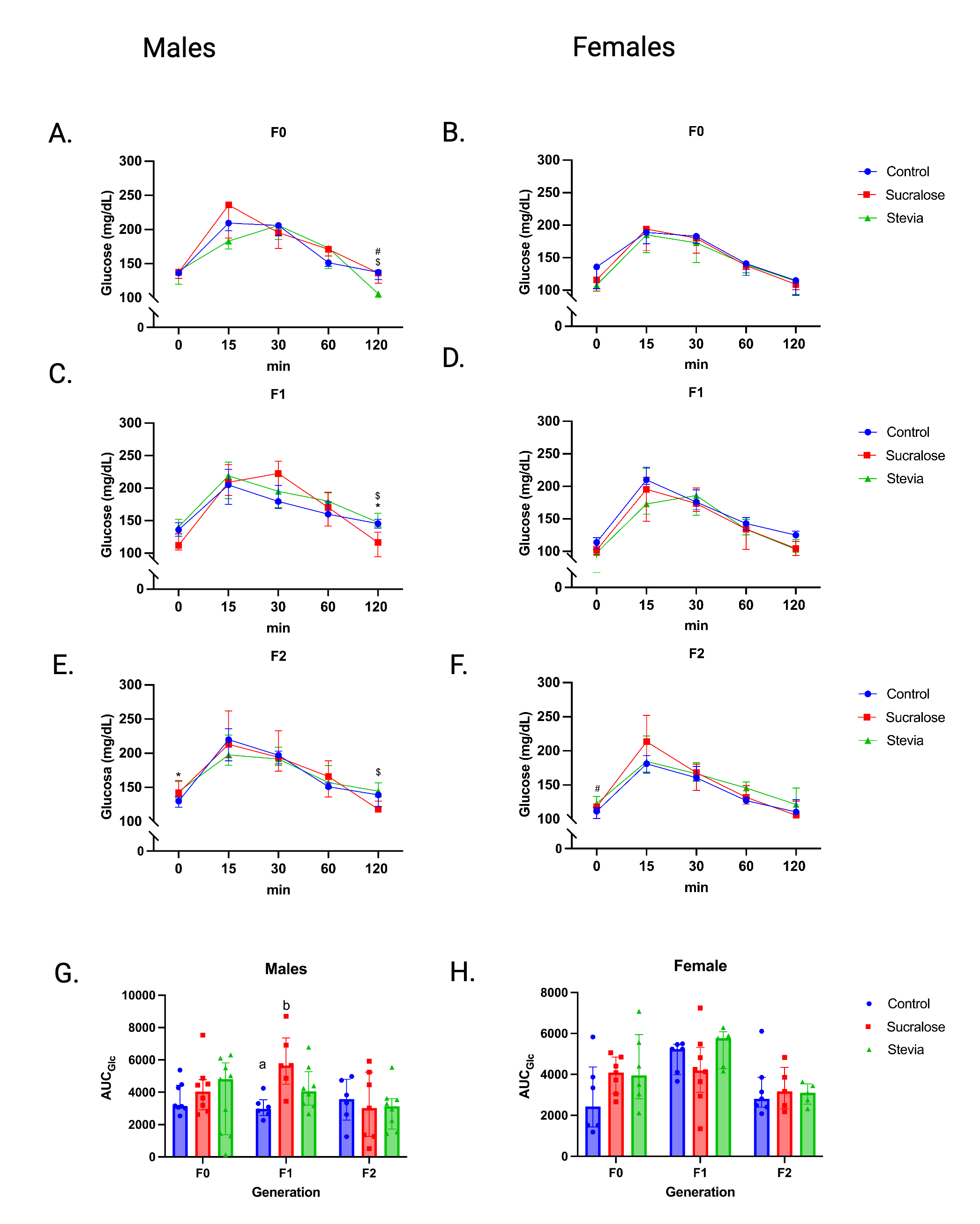
**

**Figure S1. Intergroup differences in Oral Glucose Tolerance Test by generation. G**lycemia curves of mice by generation and sex. Male (A) and female mice (B) of the F0 generation. Male (C) and female mice (D) of the F1 generation. Male ( E) and female mice (F) of the F2 generation. Area under the glycemia curve in male mice of the F0, F1, and F2 generations G). Area under the glycemia curve of female mice of the F0, F1, and F2 generations (H). (n=6-9 animals/group). Data were analyzed using the Kruskal-Wallis test, multiple comparisons using Dunn's test adjusted with the Bonferroni correction (p<0.05), results are expressed as median + interquartile range. *Significant differences between the control and sucralose groups; #Significant differences between the control and stevia groups; $Significant differences between the sucralose and stevia groups, p<0.05.

**
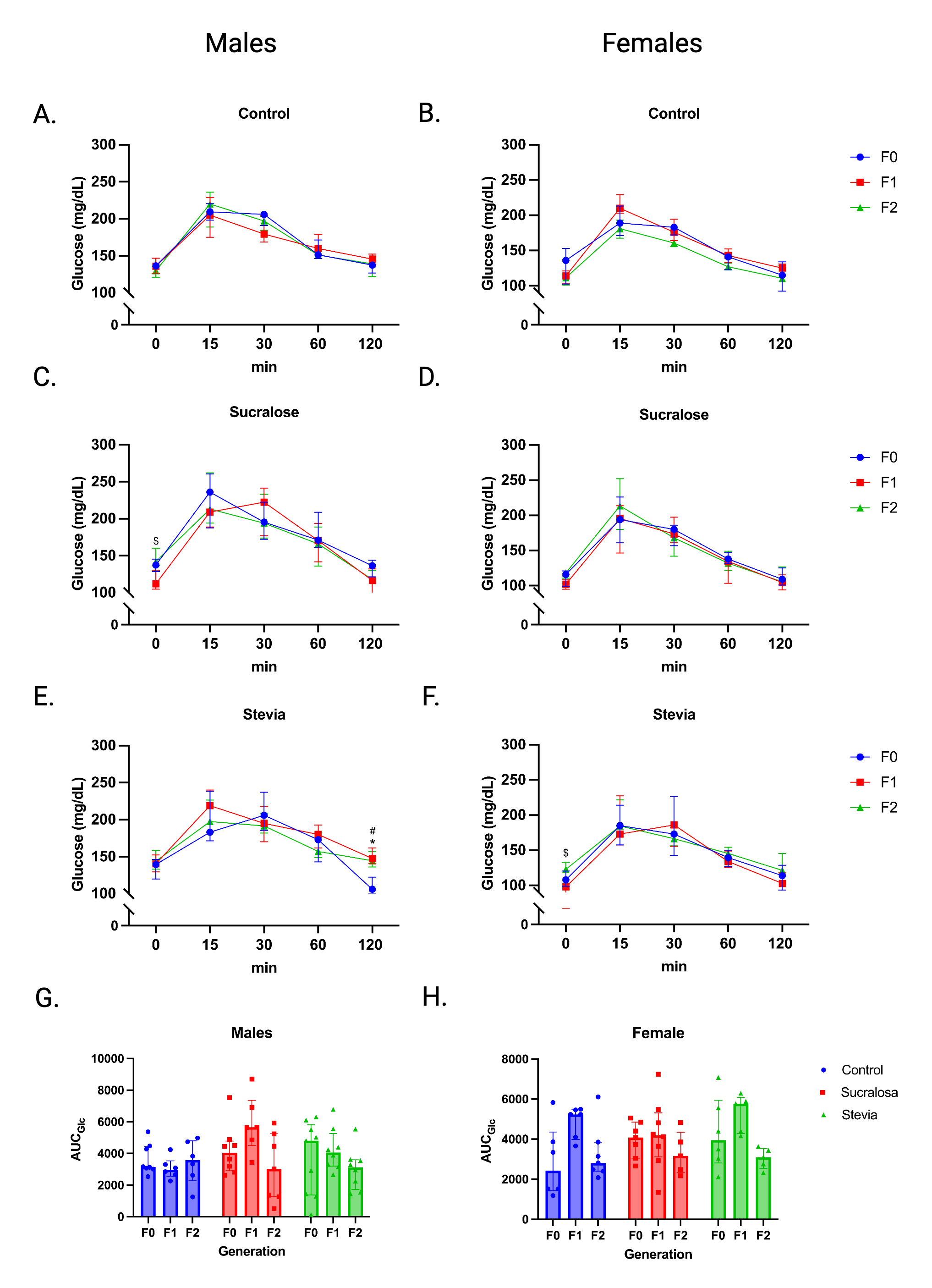
**

**Figure S2.** **Intergenerational differences in** **Oral Glucose Tolerance Test by treatment group**. Intergenerational glycemia curves of mice by group and sex. A) Male mice from the control group; B) Female mice from the control group; C) Male mice from the sucralose group; D) Female mice from the sucralose group; E) Male mice from the stevia group; F) Female mice from the stevia group; G) Intergenerational area under the glycemia curve of male mice in the control, sucralose, and stevia groups; H) Intergenerational area under the glycemia curve of female mice in the control, sucralose, and stevia groups. (n=6-9 animals/group). Data were analyzed using the Kruskal-Wallis test, multiple comparisons using Dunn's test adjusted with the Bonferroni correction (p<0.05), results are expressed as median + interquartile range. *Significant differences between F0 and F1; #Significant differences between F0 and F2; $Significant differences between F1 and F2, p<0.05.
